# Dramatic Improvement of CRISPR/Cas9 Editing in *Candida albicans* by Increased Single Guide RNA Expression

**DOI:** 10.1128/mSphere.00385-16

**Published:** 2017-04-19

**Authors:** Henry Ng, Neta Dean

**Affiliations:** Department of Biochemistry and Cell Biology, Stony Brook University, Stony Brook, New York, USA; University of Texas Health Science Center

**Keywords:** CRISPR, *Candida albicans*, RFP, double-strand-break repair

## Abstract

The clustered regularly interspaced short palindromic repeat system with CRISPR-associated protein 9 nuclease (CRISPR/Cas9) has emerged as a versatile tool for genome editing in *Candida albicans*. Mounting evidence from other model systems suggests that the intracellular levels of single guide RNA (sgRNA) limit the efficiency of Cas9-dependent DNA cleavage. Here, we tested this idea and describe a new means of sgRNA delivery that improves previously described methods by ~10-fold. The efficiency of Cas9/sgRNA-dependent cleavage and repair of a single-copy yeast enhanced monomeric red fluorescent protein (*RFP*) gene was measured as a function of various parameters that are hypothesized to affect sgRNA accumulation, including transcriptional and posttranscriptional processing. We analyzed different promoters (*SNR52*, *ADH1*, and *tRNA*), as well as different posttranscriptional RNA processing schemes that serve to generate or stabilize mature sgRNA with precise 5′ and 3′ ends. We compared the effects of flanking sgRNA with self-cleaving ribozymes or by tRNA, which is processed by endogenous RNases. These studies demonstrated that sgRNA flanked by a 5′ tRNA and transcribed by a strong RNA polymerase II *ADH1* promoter increased Cas9-dependent *RFP* mutations by 10-fold. Examination of double-strand-break (DSB) repair in strains hemizygous for *RFP* demonstrated that both homology-directed and nonhomologous end-joining pathways were used to repair breaks. Together, these results support the model that gRNA expression can be rate limiting for efficient CRISPR/Cas mutagenesis in *C. albicans*.

**IMPORTANCE** *Candida albicans* is an important human fungal pathogen. An understanding of fungal virulence factors has been slow because *C. albicans* is genetically intractable. The recent development of CRISPR/Cas in *C. albicans* (V. K. Vyas, M. I. Barrasa, G. R. Fink, Sci Adv 1:e1500248, 2015, https://doi.org/10.1126/sciadv.1500248) has the potential to circumvent this problem. However, as has been found in other organisms, CRISPR/Cas mutagenesis efficiency can be frustratingly variable. Here, we systematically examined parameters hypothesized to alter sgRNA intracellular levels in order to optimize CRISPR/Cas in *C. albicans*. Our most important conclusion is that increased sgRNA expression and maturation dramatically improve efficiency of CRISPR/Cas mutagenesis in *C. albicans* by ~10-fold. Thus, we anticipate that the modifications described here will further advance the application of CRISPR/Cas for genome editing in *C. albicans*.

## Abstract (precis)

*Candida albicans* is an important human fungal pathogen. An understanding of fungal virulence factors has been slow because *C. albicans* is genetically intractable. The recent development of CRISPR/Cas in *C. albicans* (V. K. Vyas, M. I. Barrasa, G. R. Fink, Sci Adv 1:e1500248, 2015, https://doi.org/10.1126/sciadv.1500248) has the potential to circumvent this problem. However, as has been found in other organisms, CRISPR/Cas mutagenesis efficiency can be frustratingly variable. Here, we systematically examined parameters hypothesized to alter sgRNA intracellular levels in order to optimize CRISPR/Cas in *C. albicans*. Our most important conclusion is that increased sgRNA expression and maturation dramatically improve efficiency of CRISPR/Cas mutagenesis in *C. albicans* by ~10-fold. Thus, we anticipate that the modifications described here will further advance the application of CRISPR/Cas for genome editing in *C. albicans*.

## INTRODUCTION

*Candida albicans* is an opportunistic fungus that is a major cause of fatal infections, especially in individuals with compromised immune systems. Despite its importance as a human pathogen, a deeper understanding of pathogenesis has been limited by the genetic intractability of *C. albicans*; *C. albicans* has a parasexual life cycle with no meiosis, and therefore genetic crosses are not possible. Since *C. albicans* normally exists as a diploid, recessive mutations must be made homozygous. The development of the clustered regularly interspaced short palindromic repeat system with CRISPR-associated protein (CRISPR/Cas) in *C. albicans* (1) is an important advance that promises to accelerate the pace of progress in *Candida albicans* biology research.

CRISPR/Cas is a system that provides adaptive immunity in bacteria (2–4). Adaptation of type II CRISPR/Cas for eukaryotic gene editing requires RNA-guided recruitment of the Cas9 nuclease to specific DNA sequences that are adjacent to an “NGG” protospacer adjacent motif (PAM) (5–7). Cas9 is recruited to the target site by a single guide RNA (sgRNA) comprised of a 20-nucleotide guide RNA (gRNA), which directs cleavage specificity by base-pairing complementarity to the target site, and an ~80 nucleotide *trans*-activating CRISPR-targeting RNA (tracrRNA), which binds Cas9. The sgRNA/Cas9 ribonucleoprotein is recruited to the target site, where Cas9 creates a double-strand break (DSB) 3 bases upstream of the PAM site (3). Since chromosomal breaks are lethal, there is a strong selection for their repair, either by nonhomologous end joining (NHEJ) or by homology-directed repair. The introduction of a DSB can increase the rate of recombination by repair with linear homologous DNA by several thousand-fold (5, 8, 9). Thus, donor repair fragments containing homology to regions flanking the DSB can be designed to introduce deletions or other alterations with single-base-pair precision.

Evidence has supported the idea that intracellular levels of correctly folded, nuclear localized sgRNA limit the rate of Cas9-dependent cleavage (10, 11). Up until now, *C. albicans* CRISPR/Cas studies have used the RNA polymerase III (Pol III) *SNR52* promoter (*P*_*SNR52*_) to drive sgRNA expression (1, 12). Unlike most genes transcribed by Pol III that contain internal promoters within the transcribed region, *SNR52* has an upstream promoter (13). Thus, *P*_*SNR52*_ Pol III transcripts are initiated downstream of the promoter but are not confounded by RNA polymerase II (Pol II)-associated 5′ cap and 3′ poly(A) additions that are predicted to influence sgRNA specificity and nuclear retention. *P*_*SNR52*_ has been used successfully for sgRNA expression in both *Saccharomyces cerevisiae* and *C. albicans* (1, 14), but its relative efficiency in *C. albicans* has not been compared to those of other sgRNA delivery schemes.

Our attempts to use the* P*_*SNR52*_ CRISPR system described by Vyas et al. (1) did not lead to high-efficiency mutagenesis of several *C. albicans* target genes, despite the use of different gRNAs that target different sites within each gene. Therefore, to improve CRISPR mutagenesis, we sought to optimize sgRNA expression and CRISPR/Cas efficiency in *C. albicans* by comparing different promoters and RNA processing mechanisms. Our results demonstrate that gRNA expression can indeed be rate limiting for efficient DSB cleavage in *C. albicans*, and we describe new modifications that can increase the efficiency of gene editing in *C. albicans* by 10-fold. While our major goal was to optimize gRNA expression, our experiments also revealed that in addition to homology-directed repair, under certain conditions, Cas9-dependent DSB can be repaired by nonhomologous end joining at low frequency.

## RESULTS AND DISCUSSION

### Assay system.

**FIG 1:**
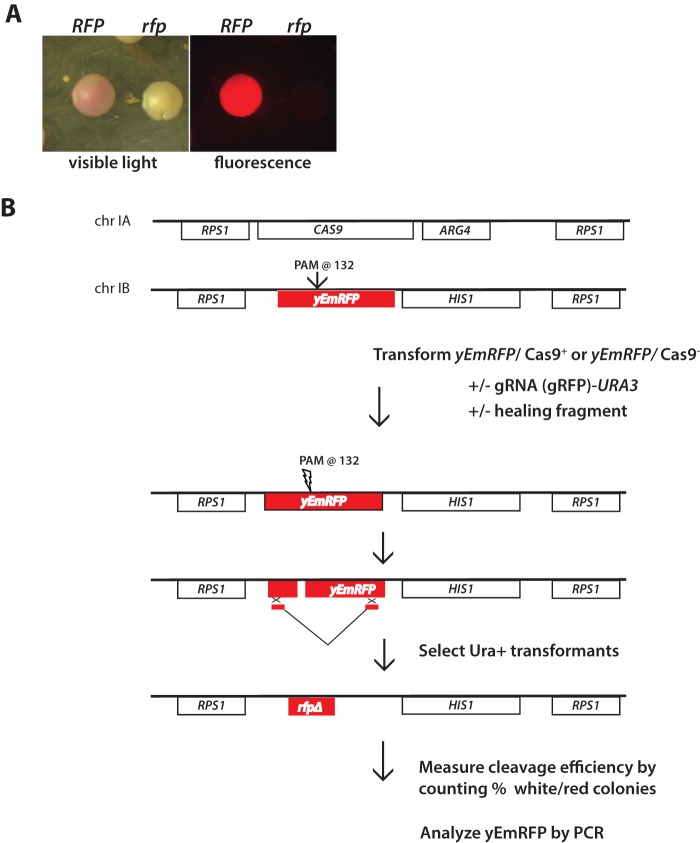
CRISPR/Cas targeting of RFP in *C. albicans*. (A) Yeast colonies that express functional *RFP* are pink and are fluorescent and can be easily distinguished from colonies that arise through CRISPR-mediated deletion of RFP, which are white and nonfluorescent. Panel B depicts the strategy for quantitating Cas9- and sgRNA-dependent cleavage *of RFP*. *RFP* (*EPC1*) or *RFP CaCAS9* (*EPC2*) hemizygous strains were constructed as described in Materials and Methods. These strains were transformed with or without a donor healing fragment and a series of *URA3*-marked plasmids that differ in expression of an RFP sgRNA (Fig. 3). The gRNA targets a 20-bp DNA sequence proximal to the *RFP* PAM site at position 132. The number of white and red colonies in each transformation was counted, and cleavage efficiency was calculated as the percentage of white colonies in the population.

To examine gRNA-dependent Cas9 nuclease activity, the *C. albicans* gene encoding codon-optimized enhanced monomeric red fluorescent protein (*yEmRFP* [henceforth referred to as *RFP*]) was targeted (15). *C. albicans* strains expressing *RFP* display a distinct pink colony color phenotype as well as bright red fluorescence (16). Inactivation of *RFP* by mutation results in a reversal of the pink colony color, red fluorescence phenotype, so colonies derived from *rfp* mutants could be easily distinguished by their white color and absence of fluorescence (Fig. 1A).

We constructed *RFP* hemizygous strains in which *HIS1*-marked *RFP* was integrated at single copy at the *RPS1* locus (Fig. 1B). As a control for Cas9-dependent *rfp* mutations, we constructed *RFP* strains that do or do not also express *CAS9*. *C. albicans* uses a noncanonical genetic code that reads the leucine CTG codon as serine. Thus, a synthetic *Candida*-optimized *CAS9* gene (*CaCAS9*) was designed to alter each CTG serine codon to an AT-rich leucine codon. In addition, the codon bias of the entire gene was maximized toward AT-rich codons (see Materials and Methods). These *RFP/CaCAS9* strains were transformed with *URA3*-marked sgRNA plasmids (described below) that targeted* RFP* cleavage proximal to the PAM site at position +132 of the *RFP* open reading frame (ORF) (Fig. 1B). The efficiency of Cas9/sgRNA-dependent cleavage was measured by determining the percentage of white, nonfluorescent *rfp* mutant colonies in the population of transformants.

**FIG 2:**
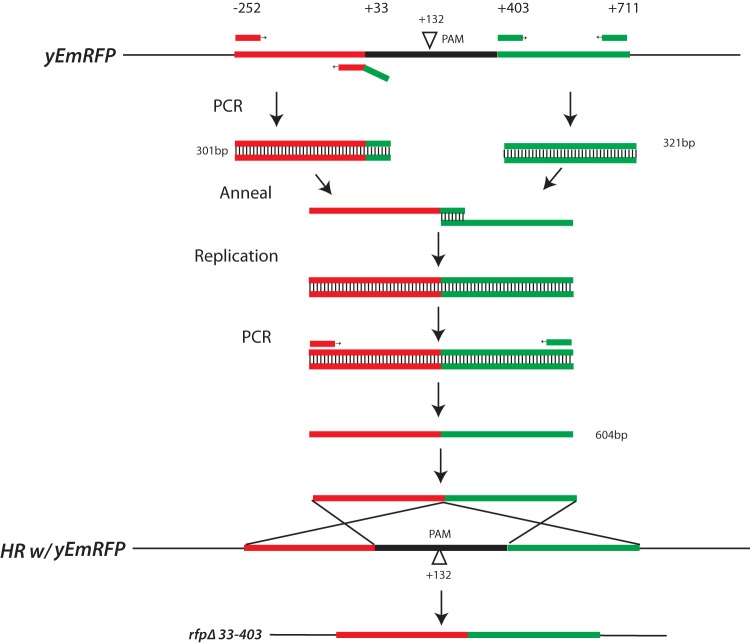
Construction of the *RFP* donor repair fragment. The donor repair fragment used for repair of the Cas9-induced DSB was constructed by overlap PCR. Two fragments were amplified by PCR, one of which contains a 3′ end that is homologous to the 5′ end of the other. After the two fragments are mixed, these homologous regions anneal to one another, and then a round of DNA replication produces a single fragment with arms of homology to the 5′ and 3′ regions of RFP, but which is deleted for 370 bp of the ORF, including the PAM site at position 132. Homologous recombination of this *rfpΔ33-403* fragment with chromosomal *RFP* can easily be detected by PCR amplification (Fig. 4).

These transformations were performed in the presence or absence of a donor repair fragment designed to delete 370 bp of the *RFP* ORF after homology-directed repair. This repair fragment was made using overlap extension PCR and contained 285-bp arms of upstream and 308-bp downstream sequence homology to regions flanking the DSB (see Materials and Methods) (Fig. 2). To confirm that* RFP* in white colonies was correctly deleted by recombination with the repair fragment, *RFP* was amplified by PCR, analyzed by gel electrophoresis for the presence of the deleted allele, and also sequenced (see below). This screen allowed a simple and accurate way to measure the efficiency of Cas9-dependent cleavage and repair of *RFP*.

### sgRNA delivery schemes.

**FIG 3:**
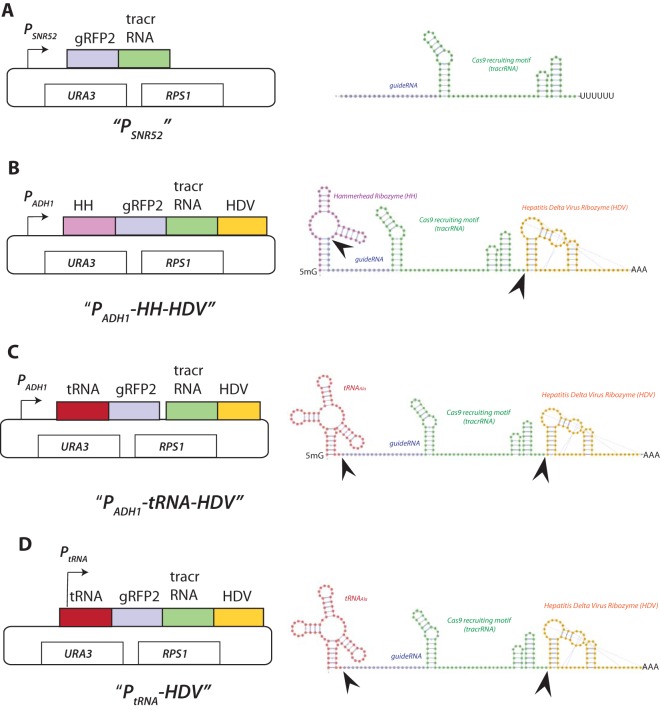
Schematic diagram of sgRNA expression cassettes and the structures of their predicted encoded RNA. The backbone of each of these plasmids is based on the *URA3* integrating CIP10 plasmid (35) (see Materials and Methods). RNA secondary structure visualization was performed using VARNY (http://varna.lri.fr/) (41). (A) *P*_***SN**R**52***_ drives transcription of the sgRNA that consists of the 20-nucleotide gRFP (purple) fused to the 85-nucleotide Cas9 recruiting tracrRNA (green). Note that this sgRNA is identical in all four delivery schemes. The gRNA has the requisite 5′ G and a 3′ poly(U) tail. (B) *P_ADH_-HH-HDV.* The sgRNA is flanked by the 5′ hammerhead (HH [pink]) and 3′ hepatitis delta virus (HDV [yellow]) ribozymes. HH-sgRNA-HDV transcription is driven by the strong *ADH1* promoter. The stem structure formed between the 5′ HH and 5′ gRNA forms the structural motif recognized for autocleavage (depicted by arrowhead). The 3′ cleavage relies on the autonomous pseudoknot structure of HDV (depicted by arrowhead). (C) *P_ADH_-tRNA*(−*HDV*). This construct is exactly like that shown in panel B, but the hammerhead sequence is replaced by the 75-bp *C. albicans* alanine tRNA gene. In this scheme, the mature 5′ end of the sgRNA is generated by endogenous RNase Z, which recognizes the stem structure formed by tRNA 5′ and 3′ sequences. 3′ cleavage relies on the autonomous stem-loop structure of HDV (depicted by arrowhead). Transcription of the tRNA-sgRNA-HDV is driven by the strong *ADH1* promoter. (D) *P*_*tRNA*_(−*HDV*). This construct is exactly like that of panel C, but the P_*ADH1*_ sequence is deleted. In this cassette, RNA Pol III transcription is driven by the internal A and B box elements of the tDNA promoter, and the mature 5′ end of the sgRNA is generated by endogenous RNase Z, while 3′ cleavage relies on the autonomous pseudoknot of HDV (depicted by arrowhead).

To optimize sgRNA expression, several parameters were varied, including the promoters used to drive transcription, as well as removable flanking sequences designed to be posttranscriptionally cleaved to produce transcripts with precise 5′ and 3′ ends. A schematic diagram of the different constructs and the predicted RNA transcripts is shown in Fig. 3, and their DNA sequences are listed in Fig. S1 in the supplemental material. Each construct had the same sgRNA sequence but differed in the promoters used to drive sgRNA transcription and/or flanking sequences that encode ribozymes or tRNAs that are posttranscriptionally cleaved.

10.1128/mSphere.00385-16.1 FIG S1  pADH/tRNA plasmids used for high-efficiency gRNA expression. (A) Schematic diagram of the CIp-P_ADH_/tRNA vector that contains a cloning cassette containing two nonpalindromic SapI sites, separated by a unique ClaI, at the tRNA-tracrRNA junction. These *RPS1* integration plasmids are marked with *URA3* (p494) or *ura3-dpl200* (p501). (B) The sequence of the SapI cassette (blue) at the tRNA (green)-tracrRNA (purple) junction. The positions of ClaI and SapI sites within the cassette are underlined, and arrows depict the SapI cleavage sites. Beneath the SapI cassette is an example of how to design two gRNA oligonucleotides (boxed, using *LEU2* gRNA). Note the gRNA (red) oligonucleotides contain CAA overhangs that create sticky ends when annealed, which allow ligation of these oligonucleotides into SapI-digested vector. Ligation results in recreation of the RNase Z tRNA recognition cleavage site, as well as the correct fusion of the gRNA with the tracrRNA. Correct ligation products are screened by the loss of the ClaI site. Download FIG S1, PDF file, 1.8 MB. Copyright © 2017 Ng and Dean. 2017 Ng and Dean This content is distributed under the terms of the Creative Commons Attribution 4.0 International license.

The first construct used the Pol III *P*_*SNR52*_ to drive sgRNA expression (*P*_*SNR52*_-*sgRNA*). The *C. albicans P*_*SNR52*_ promoter sequence and sgRNA junction were the same as those used in previous studies (Fig. 3A) (1). *P*_*SNR52*_ Pol III transcripts are initiated at a purine, G or A, and are terminated by a poly(U) stretch (six in yeast) (13).

The second delivery scheme used the strong Pol II *ADH1* promoter (*P*_*ADH1*_) to drive sgRNA transcription (Fig. 3B). Because *P*_*ADH1*_ is a strong constitutive promoter, we reasoned that *P*_*ADH1*_ may increase the levels of sgRNA. To prevent potential interference of sgRNA specificity by the 5′ cap and 3′ poly(A) tail associated with Pol II transcripts, self-cleaving hammerhead (HH) and hepatitis delta virus (HDV) ribozymes were added to the 5′ and 3′ ends of sgRNA. The presence of these ribozymes at the sgRNA ends may also protect it from nucleases and therefore further increase its intracellular levels. In the design of this cassette, the first six nucleotides of the HH ribozyme are complementary to the first six nucleotides of the gRNA, thus allowing the formation of the stem structure required for HH self-cleavage at the gRNA-HH junction (17). The sites of the HH and HDV predicted cleavage sites are indicated by the arrowheads in Fig. 3B.

To compare the efficiency of sgRNA maturation, the third delivery scheme used the same strong *P*_*ADH1*_ to transcribe sgRNA, but its 5′ end was flanked with *C. albicans* tRNA^Ala^ instead of the HH ribozyme (Fig. 3C). The rationale for this idea was 2-fold. First, endogenous RNase P and Z mediate the robust posttranscriptional cleavage of 5′ and 3′ ends of pre-tRNAs (18, 19). These endogenous RNase activities may be more efficient than HH autocatalysis and therefore lead to improved sgRNA maturation. Second, cleavage by RNase Z at the tRNA-sgRNA junction requires recognition of sequences that are only within the tRNA. Therefore, this architecture, if efficient, would provide a more portable platform for swapping out the 20-bp gRNA coding sequence with any another gRNA. This is not possible using HH ribozyme, whose unique 5′ sequence is constrained by each different gRNA. The sequence of the sgRNA-tRNA junction was designed to yield the precise sgRNA 5′ end after cleavage with RNase Z (11, 20). The sites of the tRNA and HDV predicted cleavage sites are indicated by the arrowheads in Fig. 3C.

As tRNAs are very abundant, we considered the possibility that the tRNA promoter itself may be efficient in directing sgRNA expression (11, 20). To test this, the fourth delivery scheme used the tRNA^Ala^ fused to sgRNA-HDV (Fig. 3D). Cleavage of tRNA at its 3′ trailer by endogenous RNase Z (see arrows for cleavage site) allows maturation of sgRNA with a precise 5′ end. The sites of the tRNA and HDV predicted cleavage sites are indicated by the arrowheads in Fig. 3D.

### Measuring *RFP* mutation as a function of sgRNA delivery.

**FIG 4:**
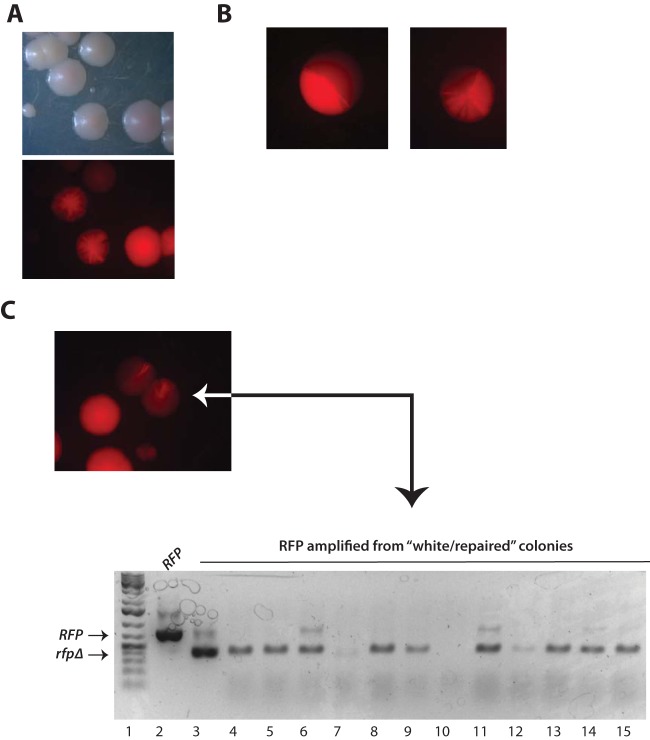
Sectored colony phenotype of *RFP* mutants. (A) Comparison of red, white, and sectored colony phenotype in visible and red fluorescent light. (B) Examples of various degrees of sectoring that range from mostly red to mostly white. (C) Red and white phenotypes in a sectored colony are genetically stable. A single sectored colony was restreaked on nonselective medium. Below is shown PCR analysis of full-length and *rfpΔ33-403* from white “repaired” colonies isolated after transformation in the presence of a donor repair fragment.

**FIG 5:**
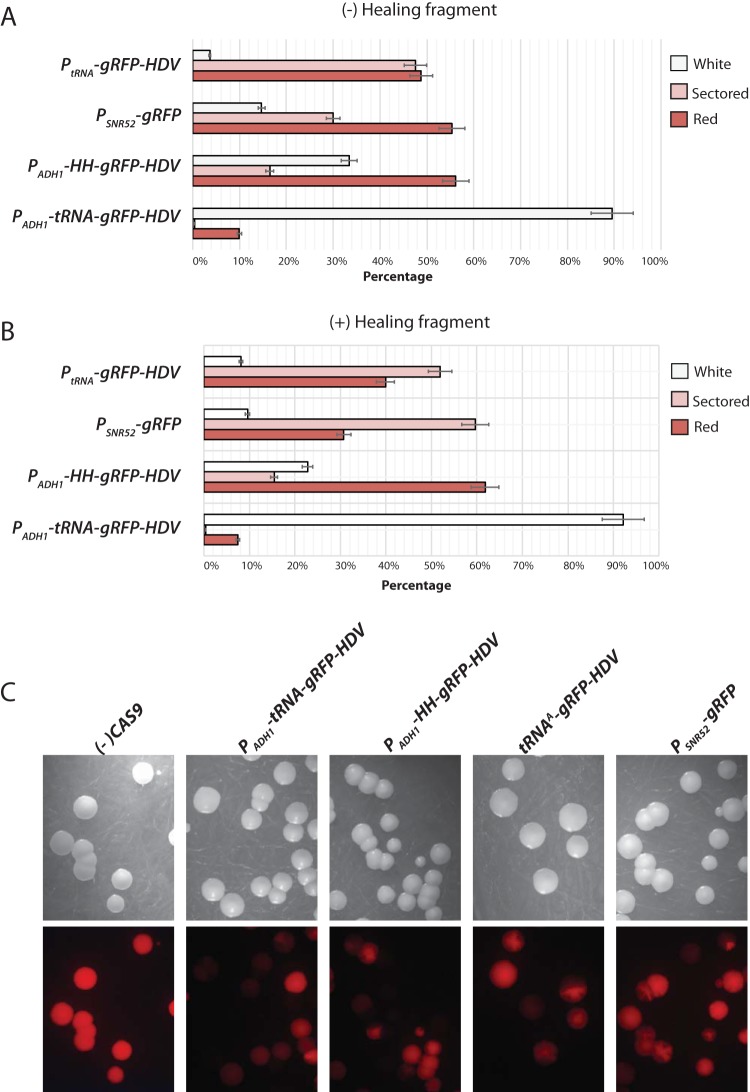
*RFP* CRISPR mutagenesis as a function of sgRNA delivery. *RFP Cas9* strain EPC2 was transformed with each of the plasmids depicted in Fig. 3. Shown are the percentages of white mutant (*rfpΔ*), red (*RFP*), and sectored colonies that arose after 2 days at 30°C. Transformations were performed in the presence of a donor homologous repair fragment (A) or in its absence (B). The data represent an average from three separate experiments in which all plasmids (plus or minus the repair fragment) were transformed side by side. Approximately 200 colonies per plate were counted and scored as red, white, or sectored. For each experiment, controls were included in which all plasmids were transformed in the isogenic strain that lacks Cas9 (not shown). (C) Light and red fluorescent images of colony phenotype as a function of different sgRNA delivery plasmids depicted in Fig. 3. Note the near absence of sectored colonies when sgRNA is efficiently delivered (*P_ADH_-tRNA-gRFP-HDV*) compared to when poorly delivered (*P*_*tRNA*_ or *P*_*SNR52*_).

To compare their relative activities, each of the *URA3*-marked CIp gRNA plasmids described above, or a negative (−) gRNA vector alone (−gRNA) was transformed side by side into* RFP* strains that did (EPC2) or did not (EPC1) express Cas9 (see Materials and Methods). Each transformation was performed in the presence or absence of a repair fragment (see Materials and Methods). After 2 days, colonies from each transformation were counted by fluorescence microscopy to determine the number of white and red colonies for each transformation. Unexpectedly, in addition to pure red and white colonies, under certain conditions, we observed red colonies with white sectors (Fig. 4). White or white-sectored colonies were seen at a frequency of less than 10^−4^ in control experiments, in which the isogenic Cas9^−^ EPC1 strain was transformed with gRNA, vector, and/or the donor repair fragment (data not shown) (Table 1). White or white-sectored colonies were also not observed in Cas9^+^ EPC2 cells transformed with vector lacking gRNA with or without the donor repair fragment. These control experiments demonstrated that the high frequency of white or white-sectored colonies was not due to spontaneous looping out of *RFP*, which is flanked by *RPS1* repeats, or by replacement of *RFP* with the CIp-gRNA plasmid during integration at *RPS1*. Instead, white and white-sectored colonies were dependent on the expression of both Cas9 and sgRNA (Fig. 5C; Table 1). Each white sector represented a Cas9-mediated *RFP* cleavage event that occurred in a cell at the vertex of the white sector. The sizes and numbers of white sectors ranged from those that were 1/2 the colony, in which RFP cleavage likely occurred in one daughter cell of the first division, to those that appear mostly white but had a remaining sliver of red cells in a colony (Fig. 4A and B). The* rfp* mutation that gave rise to white sectors was stable, since restreaking a sectored colony on –Ura plates resulted in colonies that were almost completely white (not shown). This sectoring phenotype could explain why PCR analysis of *RFP* amplified from genomic DNA from what appeared to be visually “white/healed” colonies occasionally yielded both full-length and deleted *RFP* products (Fig. 4C).

**TABLE 1:** *RFP* mutagenesis with *P_ADH_-tRNA-gRFP-HDV*

Transformation^a^	Total no. of transformants^b^	% of colonies:			% of transformants:	
		Red	White	Sectored	His^+^^c^	Arg^+^^d^
(−) Cas9 (EPC1), (+) vector (−gRFP)	~1,000	100	0	0	100	0
(−) Cas9 (EPC1), (+) gRFP	~1,000	100	0	0	99	0
(−) Cas9 (EPC1), (+) gRFP, (+) healing fragment	~1,000	100	0	0	100	0
(+) Cas9 (EPC2), (+) vector (−gRFP)	~1,000	100	0	0	100	80
(+) Cas9 (EPC2), (+) gRFP	~1,000	8	90	2	30	80
(+) Cas9 (EPC2), (+) gRFP, (+) healing fragment	~1,000	8	92	0	97	73

a Transformations were performed with *URA3*-marked *P_ADH_-tRNA-gRFP-HDV* plasmid pND482 (+gRFP) or vector lacking gRFP (−gRFP). (−), donor repair fragment absent; (+), donor repair fragment present. The results shown represent an average of 5 separate experiments, in which the percentage of white colonies ranged from 90 to 98%.

b Number of uracil prototrophic transformants.

c Percentage of uracil prototrophic transformants that were histidine prototrophs.

d Percentage of uracil prototrophic transformants that were arginine prototrophs.

Thus, to more accurately quantitate the relative efficiency of sgRNA delivery, we counted the number of white, red, and sectored colonies produced after transformation with each of the different plasmids, in the presence or absence of healing fragment. These results, shown graphically in Fig. 5A and B, demonstrated a clear hierarchy of cleavage efficiency as measured by the percentage of pure white *rfpΔ* mutant colonies in the population, in the following order: *P_ADH1_-tRNA* (~94% white)* > P_ADH_-HH* (~22% white)* > P*_*SNR52*_ (9% white)* = P*_*tRNA*_ (~8% white). When sgRNA transcription was driven by the Pol II* ADH1* promoter but flanked by the 5′ hammerhead ribozyme instead of the tRNA, the Cas9-directed mutation frequency was reduced by ~3.5-fold. When sgRNA transcription was driven by the Pol III *SNR52* or *tRNA* promoters, the mutation frequency was reduced by ~10-fold. While the experiments shown in Fig. 5 were performed using CIp-based integration plasmids, the same trend was observed using *CaARS*-containing plasmids (data not shown). Therefore, we inferred that the high efficiency observed when sgRNA was delivered using the *P_ADH1_-tRNA* plasmid is due to the combination of (i) increased Pol II *P*_*ADH1*_ promoter strength, compared to the Pol III *P*_*SNR52*_ or *P*_*tRNA*_, and (ii) the presence of the tRNA flanking sequence. These results support the idea that the presence of the tRNA may promote sgRNA nuclear retention or stabilization and in addition is processed by endogenous RNase Z more efficiently than HH autocatalysis. An additional factor that may explain the reduced efficiency of transcripts driven by *P*_*SNR52*_ is that the sgRNA is constrained by a 5′ purine, which is the favored nucleotide for transcription initiation by *P*_*SNR52*_. A non-base-paired 5′ G, which is built into the cloning site of *P*_*SNR52*_ CRISPR vectors, may result in 5′ overhang in the sgRNA-target DNA complex that could impact sgRNA efficiency, although this has not been formally tested. Thus, of the sgRNA delivery schemes we tested, the *P*_*ADH1*_*-tRNA*-driven sgRNA expression was the most efficient, by almost 10-fold.

Quantitation of sectored colonies demonstrated a strong correlation between the abundance of sectored colonies and the inefficiency of the sgRNA delivery. In a population of *P_ADH1_-tRNA* transformants, over 90% of colonies were white, while less than 1% were sectored. Conversely, in a population of *P*_*tRNA*_ transformants, less than 10% of colonies were white, while more than 50% were sectored (Fig. 5C). These results are shown quantitatively in Fig. 5. One interpretation of these results is that efficient Cas9-dependent cleavage requires a threshold level of sgRNA that is not met in founder transformants when sgRNA is poorly expressed. After one or more cell divisions, daughter cells may eventually accumulate sufficient levels of sgRNA to allow Cas9 DSB and repair.

### RFP DSB repair.

**FIG 6:**
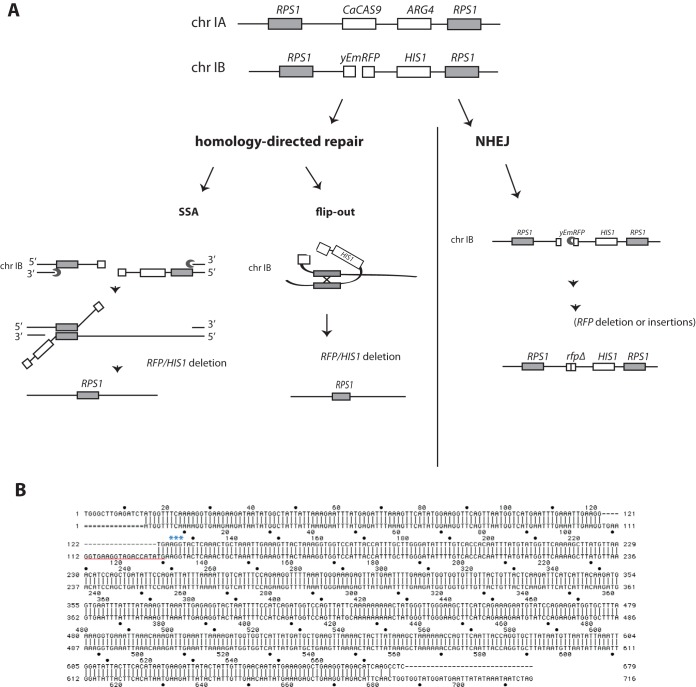
Models for DSB repair at the hemizygous *RFP* locus by homology-mediated repair and NHEJ. (A) Schematic diagram of homology-mediated and NHEJ pathways of DSB repair in *RFP* in the absence of donor repair fragment. A variety of homology-directed repair pathways between duplicated *RPS1* loci are possible. Both flip-out and single-strand annealing (SSA) are consistent with the observed simultaneous loss of both *RFP* and *HIS1* when a DSB is introduced in* RFP*. (B) Alignment of the wild-type *RFP* sequence with that of RFP amplified from a white colony isolated after CRISPR mutagenesis in the absence of a donor repair fragment. The sequence targeted by the *RFP* gRNA is underlined in red and precedes the PAM site (AGG, denoted with blue asterisks) at *n =* 132. Note that the deletion begins 3 bases from the PAM, precisely at the predicted Cas9 cleavage site.

Surprisingly, we observed that the relative numbers of white, red, and sectored colonies for each of the different sgRNA plasmids were similar whether or not a donor repair fragment was included in the transformation (compare Fig. 5A and B). These results raised the question of how breaks in *RFP* were repaired in the absence of homologous recombination with the repair fragment. The strain used for these experiments is hemizygous for *RFP*. Since this sequence is not repeated anywhere else in the genome, this DSB cannot be repaired by gene conversion. However, *RFP* is flanked by a duplication of *RPS1* loci (see Materials and Methods) (Fig. 1 and Fig. 6A), and there are additional duplicated* RPS1* loci that were introduced by integration of the gRNA plasmid, as well as *CAS9*. Thus, Cas9-mediated DSB in *RFP* could stimulate its deletion by a variety of homology-directed pathways that involve recombination between adjacent *RPS1* loci (i.e., by flip-out or single-strand annealing as depicted in Fig. 6A) (21) or even* RPS1* on the homologous chromosome (Fig. 6A). These types of homology-directed repair pathways would lead to deletion of *RFP* sequence, which includes *RFP* and *HIS1*. An alternative means of DSB repair is nonhomologous end joining (NHEJ), in which the breaks are directly ligated, although this is usually accompanied by short insertions or deletions (Fig. 6A) (22, 23).

To investigate how these DSB were repaired in the absence of donor healing fragment, *RFP* from these white colonies (~30) was amplified by PCR, analyzed by gel electrophoresis, and subjected to DNA sequencing. If DSBs at *RFP* were repaired by homologous recombination between adjacent *RPS1* loci, then both the *RFP* and *HIS1* genes should be completely deleted. To test this, these colonies were also replica plated on −His plates to determine the presence or absence of *HIS1*. The result of this experiment demonstrated that by PCR analysis, 70% of these white colonies lacked the *RFP* gene entirely and were also histidine auxotrophs, suggesting that in these colonies, *RFP* DSB repair occurred via a homology-directed repair pathway (Fig. 6A).

The remaining 30% of these white colonies contained *RFP* of a similar electrophoretic mobility as the full-length gene. As exemplified in Fig. 6B, the alignment of wild-type *RFP* with one such mutant showed the presence of a 21-bp deletion that removes the PAM site and sgRNA targeting sequence (Fig. 6B [GGTGAAGGTAGACCATATGAA represents the gRNA sequence underlined in red]). The mutant DNA sequence (shown in Fig. 6C and Fig. S2 in the supplemental material) indicates that the Cas9 cut site occurred 3 bases from the PAM (AGG at position 132, depicted by blue asterisks in Fig. 6B). This result suggested that repair occurred via nonhomologous end joining, resulting in a short deletion. This was a surprising result because several studies have demonstrated that, in *C. albicans* as in *S. cerevisiae*, homologous recombination is a vastly preferred mechanism for repair of DSB since mutations that knock out homologous recombination affect the sensitivity of strains to agents that produce DSB, while mutations in NHEJ do not (24, 25). Our results suggested that although homology-directed repair is the predominant pathway, DSBs can be repaired in *C. albicans* by NHEJ.

10.1128/mSphere.00385-16.2 FIG S2  NHEJ DNA sequence trace. Shown is a partial DNA trace of an *rfp* mutant allele whose sequence is consistent with an NHEJ event. A schematic diagram of a portion of the sequence aligned to RFP is depicted, showing deletion endpoints. (See the sequence alignment in Fig. 6B.) Download FIG S2, PDF file, 0.2 MB. Copyright © 2017 Ng and Dean. 2017 Ng and Dean This content is distributed under the terms of the Creative Commons Attribution 4.0 International license.

### Extension of system to endogenous genes.

The results described above demonstrated a system for efficient targeting and repair of *RFP*. However, *RFP* was present in the chromosome as single copy and flanked by direct repeats, which may affect its mutability. Therefore, to confirm the utility of the *P_ADH1_-tRNA* sgRNA delivery scheme for deletion of endogenous diploid genes, we constructed *URA3-* or recyclable *ura3-dpl200* (26)-marked *P_ADH_-tRNA* vectors (pND494 and pND501) to allow optimized expression of any gRNA. These plasmids allow ligation of short (23-bp) annealed oligonucleotides into a cloning cassette containing two nonpalindromic SapI sites at the tRNA-gRNA junction (see Materials and Methods and Fig. S1). This cassette was designed to maintain the RNase Z cleavage recognition site at the tRNA-gRNA junction as well as the seamless fusion of gRNA to tracrRNA after ligation of annealed gRNA oligonucleotides (Fig. S1).

**FIG 7:**
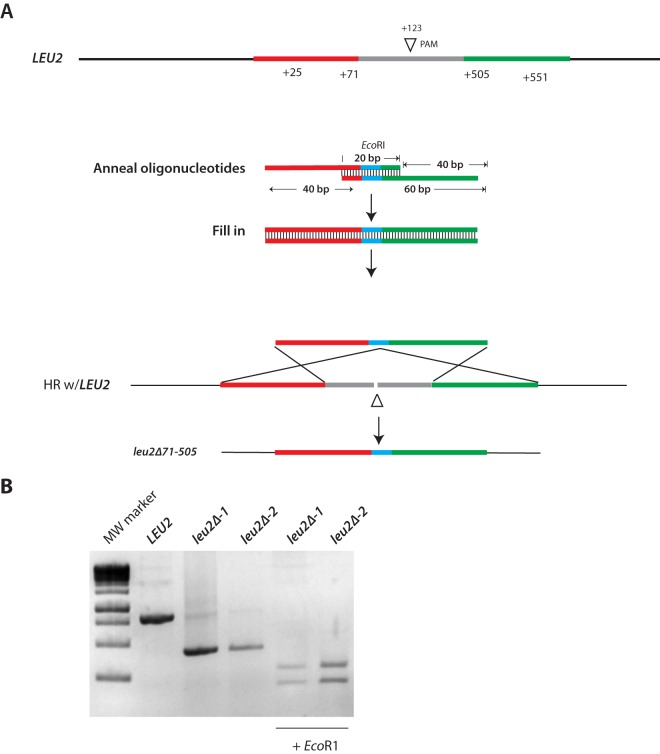
Markerless deletion of *LEU2*. (A) Schematic diagram of the *LEU2* locus, showing the PAM site at position 123. Sequences homologous to the repair fragment are colored in green and red. Each 60-mer oligonucleotide ends with a 20-bp sequence of complementarity, including a restriction site that is absent in *LEU2* (EcoRI), shown in blue. When annealed and extended, this repair fragment contains 47 bp of homology to sequence flanking the DSB. Homologous recombination (HR) results in a 434-bp deletion of LEU2, which is replaced by a unique EcoRI site. (B) PCR analysis of DNA from *LEU2* and *leu2Δ* mutants isolated by CRISPR (see text). EcoRI digestion of PCR products verified the *leu2* deletion genotype. MW, molecular weight.

As proof of principle, we targeted *LEU2* with a gRNA complementary to the region proximal to the PAM at position +123 of the *LEU2* ORF (Fig. 7). A Cas9^+^ strain (HNY30) was transformed with a *LEU2*-specific gRNA plasmid marked with *URA3* or vector lacking gLEU2 (Table 2). These transformations were performed in the absence or presence of a donor repair fragment designed to replace a 434-bp fragment within the* LEU2* ORF with a unique EcoRI site (see Materials and Methods) (Fig. 7A). This repair fragment contained 47-bp arms of homology to regions flanking the break. As noted by Vyas et al. (1), we found that repair fragments with arms of longer homology (80, 250, or 500 bp) did not improve the efficiency of mutagenesis (not shown). Controls included the same transformations but of a Cas9^−^ isogenic strain (BWP17). To quantify mutations in *LEU2*, Ura^+^ transformants were patched on plates containing synthetic dropout medium without uracil [SD(−Ura)] and then replica plated on SD(−Ura) and SD(−Leu) to identify leucine auxotrophs (Table 2). *LEU2* from both Ura^+^ and Leu^−^ transformants was genotyped by PCR (Fig. 7B; Table 2).

**TABLE 2:** *LEU2* mutagenesis

Transformation^a^	No. of transformants:		% of:	
	Ura^+^	Leu^−^^b^	*LEU2*/*leu2* heterozygotes^c^	*leu2Δ*/*leu2Δ* homozygotes^d^
(−) Cas9, (+) vector	620	0	0	0
(−) Cas9, (+) gLEU2	700	0	0	0
(−) Cas9, (+) gLEU2, (+) repair fragment	650	0	0	0
(+) Cas9, (+) gLEU2	28	0	0	0
(+) Cas9, (+) gLEU2, (+) repair fragment	760	608	0	80

a Transformations were performed with BWP17 (Cas9^−^) or HNY30 (Cas9^+^) with *URA3*-marked* LEU2* gRNA plasmid, p499 (+gLEU2), or the vector that lacks gRNA (p494). (−), donor repair fragment absent; (+), donor repair fragment present. The results shown represent the average from 3 experiments.

b The number of Ura^+^ transformants that were leucine auxotrophs.

c The percentage of *LEU2*/*leu2Δ* heterozygotes was based on PCR analysis of *LEU2* using genomic DNA isolated from 40 random Ura^+^/Leu^+^ transformants.

d The percentage of *leu2Δ*/*leu2Δ* homozygotes was based on PCR analysis of *LEU2* amplified from genomic DNA isolated from 60 random Ura^+^/Leu^−^ transformants.

The results of these experiments revealed some important differences between *LEU2* and *RFP* mutagenesis. First, in contrast to what was observed with RFP, the number of Ura^+^ gLEU2 transformants that were obtained in the absence of healing fragment was ~25-fold lower than in its presence. This reduction in transformation was dependent on the presence of both Cas9 and *LEU2*-specific gRNA, suggesting it was caused by DSBs (Table 2). Second, in the absence of healing fragment, none of the viable Ura^+^ transformants were leucine auxotrophs (Table 2). Thus, a plausible explanation for this reduction is that donor repair fragment is required to repair breaks in *LEU2*, and that unrepaired breaks are lethal. This implies that other non-homology-directed repair pathways, including NHEJ, occur very rarely. Third, in the presence of healing fragment, ~80% of Ura^+^ transformants were Leu^−^. PCR analyses of these Leu^−^ mutants suggest that all of them arose through homologous recombination with the donor repair fragment (Table 2; Fig. 7B). Finally, from random PCR analysis of Ura3^+^ transformants, we found no evidence for heterozygosity, since PCR amplification products from single colonies yielded either wild-type or truncated (EcoRI-sensitive) *LEU2*, but not both (Table 2; data not shown). The lack of heterozygosity was not specific for *LEU2* since similar results were obtained for other nonessential endogenous genes that were targeted for mutagenesis (data not shown), including *ANP1*, *VAN1*, and *BMT1*, whose further characterization will be described elsewhere.

These experiments demonstrate the applicability of this expression system for creating DSBs in endogenous diploid genes with high efficiency. This system has several advantages compared to others that have been described (1, 27). First, the higher mutation frequency, through use of the *ADH1* promoter and tRNA instead of the *SNR52* Pol III promoter, means that fewer yeast colonies require screening in order to identify a desired mutation. Second, while the transient system described by Min et al. (12) is very simple, we found that even under optimal gRNA expression, the mutation frequency is too low for mutagenesis without selection of a marker-linked donor repair fragment. The expression system described in the present study facilitates “scarless” mutations, although it requires selection of marker-linked gRNA. gRNA can be linked to *URA3* as described here or nourseothricin resistance as described by Vyas et al. (1). The advantage of uracil selection is that it is faster and cheaper than Nat^r^. Also, certain mutations—for instance those that strongly interfere with cell wall biosynthesis—display altered nourseothricin sensitivities (our unpublished observation), which can complicate their isolation using Nat^r^ selection. On the other hand, recycling *ura-dpl200* requires counterselection with 5-fluoroorotic acid (FOA), which may bias toward selection of aneuploidy (28). A basic protocol for the day-to-day timeline for a single-gene knockout using the vectors described in this study is outlined in Fig. S3 in the supplemental material.

10.1128/mSphere.00385-16.3 FIG S3  Timeline for CRISPR/Cas mutagenesis in *C. albicans*. Download FIG S3, PDF file, 0.1 MB. Copyright © 2017 Ng and Dean. 2017 Ng and Dean This content is distributed under the terms of the Creative Commons Attribution 4.0 International license.

### Concluding remarks.

Genetic analysis of *C. albicans* has been complicated because it is a diploid that does not readily undergo sexual reproduction. Without CRISPR, genetic modifications, including knockouts, must be applied to both chromosomes, requiring sequential modification of each locus. The application of the CRISPR/Cas system as described by Vyas et al. (1) has enormous potential because it can circumvent these problems. However, as has been found in other systems, the efficiency of CRISPR/Cas can be frustratingly variable. Studies from other systems suggest that several factors influence efficacy of CRISPR/Cas, including (i) location and accessibility of gRNA target site, (ii) gRNA sequence, and (iii) sgRNA intracellular levels. Here, we systematically examined parameters hypothesized to alter sgRNA intracellular levels in order to optimize CRISPR/Cas in *C. albicans*. Our most important conclusion is that increased sgRNA expression and maturation dramatically improve efficiency of CRISPR/Cas mutagenesis in *C. albicans*. Large-scale analyses of the sgRNA target site effects, chromatin structure, and gRNA sequence preferences have led to an increasing knowledge base, as well as online tools that help design gRNAs. Features of a “good” gRNA include guanines at the −1 and −2 positions (i.e., a 3′ GG) and cytosine at the −3 DNA cleavage site and at +1 relative to the N_0_G_0_G_0_ PAM site (29–32). Features of a good target position appear to be nucleosome-free locations upstream of transcriptional start sites (29–31, 33, 34). It is notable that neither the gRFP nor its target location used in the present study conforms to any of these predictive guides, yet when optimized for expression, it nevertheless resulted in an almost 100% mutation frequency. These results suggest that in *C. albicans*, sgRNA levels may in part compensate for a less than optimal gRNA design. Thus, we anticipate that the modifications described here will further advance the application of CRISPR/Cas for genome editing in *C. albicans*.

## MATERIALS AND METHODS

### Plasmid construction.

The plasmids and their relevant features are listed in Table 3, and the sequence of each relevant cassette and the oligonucleotides used for the construction are listed in Table S1 in the supplemental material. All DNA generated by PCR was verified by DNA sequence analysis.

10.1128/mSphere.00385-16.4 TABLE S1  DNA sequence of plasmids, gRNA, and expression cassettes used in this study. sgRNA and promoters are color coded according to Fig. 3. Download TABLE S1, PDF file, 0.1 MB. Copyright © 2017 Ng and Dean. 2017 Ng and Dean This content is distributed under the terms of the Creative Commons Attribution 4.0 International license.

**TABLE 3:** Plasmids used in this study

Plasmid no.	Name	Relevant feature(s)	Reference/source
pND294	CIp10	*CaURA3 RPS1* integrative plasmid	42
pND500	CIp-His1	*CaHIS1 RPS1* integrative plasmid	16
pND383	CIp-Arg4	*CaArg4 RPS1* integrative plasmid	This study
pND442	YPB1-ADHp	*CaADH1* promoter/*URA3*/*C. albicans* ARS	35
pND354	CIpHis1-P_ADH1 _yEmRFP	*yEmRFP* driven by *CaADH1* promoter in CIp-His	16
pND425	CIpArg4-P_TEF _CaCas9	Codon-optimized *Cas9* driven by *AgTEF1* promoter in CIp-Arg	This study
pND459	YPB-P_ADH1 _HH gRFP HDV	*P_ADH1_HH gRFP HDV* in YPB	This study
pND465	YPB-P_ADH1 _HH gLeu2 HDV	*P_ADH1_HH gLeu2 HDV* in YPB	This study
pND468	YPB-P_ADH1 _tA gRFP HDV	*P_ADH1_tRNA gRFP HDV* in YPB	This study
pND479	YPB-P_tRNA_ gRFP HDV	*P*_*tRNA*_* gRFP HDV* in YPB	This study
pND474	pV1090	*P*_*SNR52*_-gRNA/*SAT*^R^ integrative plasmid	1
pND476	pV1090-gRFP	*P_SNR52_-gRFP* in pV1090	This study
pND489	pV1025	CaCas9/*SAT* flipper *ENO1* integrative plasmid	1
pND486	CIp10-P_ADH1_ HH gRFP HDV	*P_ADH1_HH sgRFP HDV* in CIp10	This study
pND482	CIp10-P_ADH1_ tA gRFP HDV	*P_ADH1_tRNA sgRFP HDV* in CIp10	This study
pND483	CIp10-P_tRNA_ gRFP HDV	*P*_*tRNA*_* sgRFP HDV* in CIp10	This study
pND484	CIp10-P_SNR52_ gRFP	*P*_*SNR52*_* sgRFP* in CIp10	This study
pND494	CIp10-P_ADH1_ tA SapI HDV	CIP10-based cloning vector for ligation of gRNA *P*_*ADH1*_*tA*-*Sap*I2× *HDV* in CIp10	This study
pND499	CIp10-P_ADH1_ tA gLEU2 HDV	*P_ADH1_tRNA sgLeu2 HDV* in CIp10	This study
pND501	CIp-dpl-P_ADH1_ tA SapI HDV	pND494 but contains *ura3-dpl200* instead of *URA3*	This study

CIp-His1-P_ADH1_ yEmRFP (pND354) is a *HIS1* integration plasmid that contains the *C. albicans* codon-optimized *yEmRFP* (15). CIp-ARG4 (pND383) was constructed by replacing the *URA3* gene in CIp10 (35) with a 1-kb BamHI/SacI fragment containing *C. albicans ARG4*. Unless otherwise noted, CIp plasmids were linearized with StuI to target integration at *RPS1*.

A *C. albicans* codon-optimized* CAS9* gene, encoding CaCas9 endonuclease with a hemagglutinin (HA) epitope tag and nuclear localization signal at the C terminus and driven by the strong *Ashbya gossypii TEF1* promoter, was synthesized (Genescript, NJ) and cloned into CIp-ARG4 as a KpnI/SacI fragment to produce CIp-ARG4-P_TEF_ CaCAS9 (pND425). YPB1-ADHp (pND442) is a 2µ *CaURA3 CaARS* plasmid that contains the *ADH1* promoter and terminator (35).

YPB-P_ADH1_ HH gRFP HDV (pND459) contains an sgRNA that targets the *RFP* PAM at position +132. sgRNA expression is driven by the RNA polymerase II (Pol II) *ADH1* promoter (*P*_*ADH1*_); the 5′ and 3′ ends are flanked by the self-cleaving hammerhead (HH) (36) and hepatitis delta virus (HDV) (37) ribozymes, respectively. A 229-bp BglII/MluI fragment (Table S1) containing the HH-gRFP-HDV sequence was synthesized and cloned into the BglII/MluI fragment of YPB1-ADHp, downstream of *P*_*ADH1*_.

YPB-P_ADH1_ HH gLeu2 HDV (pND465) is identical to YPB-P_ADH1_ HH gRFP HDV but contains a gRNA that targets the *CaLEU2* PAM site at position +123 and an HH sequence whose 6 nucleotides at the 5′ end are complementary to the first 6 nucleotides of gLeu2 (Fig. S1). The HH-gLeu2-HDV cassette was synthesized (Genescript, NJ), and cloned into YPB1-ADHp vector as a BglII/MluI fragment.

YPB-P_ADH1_ tA gRFP HDV (pND468) contains the 75-bp *C. albicans tRNA*^*Ala*^ gene between the *ADH1* promoter and the sgRNA in YPB1-Adhp. The *tRNA* gene was amplified by PCR from genomic DNA. Gibson assembly (38) was used to assemble the tRNA-sgRNA-HDV fragments and the YPB-ADHp vector.

YPB-P_tRNA_ gRFP HDV (pND479) was generated by deleting the *ADH1* promoter of YPB-P_*ADH1*_ tA gRFP HDV (pND468) by digestion with NotI and BglII, filling in overhangs with Klenow DNA polymerase and ligating.

To construct the CIp10 series of sgRNA delivery plasmids, each of the sgRNA cassettes described above (P_ADH1_ HH gRFP-HDV [1,113 bp], P_ADH1_ tA gRFP-HDV [1,147 bp], and P_tRNA_ gRFP HDV [546 bp]) was cloned into CIp10 as SalI/MluI fragments to produce pND486, pND482, and pND483, respectively. To construct CIp10-P_SNR52_ gRFP, the gRFP gRNA was first cloned into pV1025 as described previously (1). The 1,112-bp P_SNR52_ gRFP-tracr fragment was then amplified by PCR using primers with SalI and MluI sites and inserted into CIp10 to produce CIp10-P_SNR52_ gRFP (pND484).

CIp10-P_ADH1_ tA SapI HDV (pND494) is a vector that allows cloning and expression of any gRNA such that the sgRNA transcript is flanked with 5′ tRNA and 3′ HDV and transcribed by *P*_*ADH1*_. It was constructed by using site-directed mutagenesis to replace the RFP gRNA segment in CIp10-P_*ADH1*_ tA gRFP HDV with a cassette containing two SapI sites and 22 bp of intervening sequence, including a ClaI site (Fig. S2). In addition, site-directed mutagenesis replaced the unique SapI site at position 4381 of CIp10-P_ADH1_ tA gRFP HDV with an NsiI site (Fig. S2). It should be noted that while constructing this plasmid, we discovered that the CIp10 sequence (GenBank accession no. AF181970) between KpnI and SacI (containing pBluescript KS+ sequence) was incorrectly annotated and actually flipped, which places the T7 promoter adjacent to *RPS1* and the T3 promoter adjacent to *CaURA3*. To allow sequential introduction of additional gRNAs into the same strain, we also constructed a plasmid that is identical to pND494 but contains the recyclable *ura3-dpl200* allele (26). This *URA3* is flanked by 200-bp repeats that facilitate its recycling by FOA selection.

### Strains and growth conditions.

*C. albicans* strains were grown in standard rich YPAD medium (2% Bacto-peptone, 2% dextrose, 1% yeast extract, 20 mg/liter adenine) or synthetic dropout (SD) (2% dextrose, 2% Difco yeast nitrogen base with ammonium sulfate) supplemented with the appropriate nutritional requirements. Uridine (75 mg/liter) was added to all media except SD(−Ura).

The *C. albicans* strains used in this study are listed in Table 4 and were derived from BWP17. EPC1 contains a single integrated copy of the *RFP* gene (16) and was constructed by targeting StuI-linearized *CIp-HIS-P_ADH_-RFP* to *RPS1*. This integration results in a duplication of *RPS1* flanking *P_ADH1_-RFP*, *HIS1* and the intervening plasmid sequence. Single integration of *P_ADH1_-RFP* at *RPS1* was confirmed by Southern blotting (not shown). EPC2, which expresses both *RFP* and *CaCAS9*, was constructed by targeting *CIp-ARG-P_TEF1_-CaCas9* (see below) in a second round of integration into the second *RPS1* allele in EPC1. An isogenic strain, RJY54 that expresses *CaCAS9* but not* RFP*, was constructed by targeting *CIp-ARG-P_TEF1_-CaCas9* (see below) to *RPS1*. HNY30 (*eno1Δ*::*Cas9*) was constructed by targeting integration of the KpnI/SacI Cas9/SAT-flipper cassette of pV1025 (1) in BWP17 and then plating *SAT*^R^ colonies on YPAD to screen for *SAT*^S^ strains that lost the *SAT*-flipper cassette. HNY25 was constructed by knocking out *LEU2* in RJY54 using the *URA3*-marked CRISPR/Cas gLEU2 plasmid p465. After confirming the homozygous *leu2* mutation by PCR, uracil auxotrophs were selected on plates containing 5-fluoroorotic acid (FOA) and further screened for spontaneous loss of *CIp-ARG-P_TEF1_-CaCas9* by arginine auxotrophy. HNY31 (*leu2Δ/leu2Δ eno1Δ*::*Cas9*) was constructed by targeting integration of the KpnI/SacI Cas9/SAT-flipper cassette of pV1025 in HNY25 and screening for *SAT* sensitivity.

**TABLE 4:** Strains used in this study

Strain	Genotype	Reference
BWP17	*ura3Δ*::*λ imm434*/*ura3Δ*::*λ imm434 his1Δ*::*hisG*/*his1Δ*::*hisG arg4Δ*::*hisG*/*arg4Δ*::*hisG*	27
EPC1	BWP17 *RPS1*::*P*_*ADH1*_* RFP-HIS1 RPS1*	This study
EPC2	BWP17 *RPS1*::*P*_*ADH1*_* RFP-HIS1-RPS1*::*P*_*TEF1*_* CaCas9-HA-ARG4-RPS1*	This study
RJY54	BWP17 *RPS1*::*P*_*TEF1*_* CaCas9-HA-ARG4-RPS1*	This study
HNY25	BWP17 and *leu2Δ*/*leu2Δ*	This study
HNY30	BWP17 *eno1Δ*::*CaCas9*	This study
HNY31	BWP17 *eno1Δ*::*CaCas9 leu2Δ*/*leu2Δ*	This study

### Yeast transformation and quantitation of *RFP* cleavage.

Yeast transformations were performed by the lithium acetate protocol (39) with the following modifications. Fresh overnight cultures (12 to 16 h) were diluted 1:20 and incubated for ~5 h (optical density at 600 nm [OD_600_] of 5). Five milliliters was harvested, washed once with H_2_O and once with 100 mM lithium acetate (LiOAc), and resuspended in 500 µl 100 mM LiOAc. The concentration of plasmid DNA was titrated to yield ~200 colonies per plate. Typically, transformations included 50 µl of the cell suspension (2.5 OD_600_ units), ~2 to 4 µg of linearized sgRNA plasmid (1 pmol), 1 to 2 µg of RFP donor repair DNA fragment (15 pmol), or annealed, filled-in repair oligonucleotides (200 pmol). After transformation, plates were incubated at 30°C for 2 days before being viewed by fluorescence microscopy with a Zeiss microscope equipped with low-power magnification (1.5 to ×10). Visual detection of pink (*RFP*) and white (*rfp*) colonies required 3 days of incubation. The number of red, white, and sectored colonies per plate was counted to determine the efficiency of Cas9-mediated cleavage. Cleavage efficiency was calculated as the no. of white colonies/total no. of colonies per plate.

### PCR analysis of Cas9-mediated cleavage.

For analyses of *RFP* mutagenesis, white colonies were patched and replica plated on selective media to test for prototrophy of selective markers (*URA3*, *HIS1*, and *ARG4*). Genomic DNA from 20 white colonies per experiment was prepared and used as the template for PCR amplification using primers specific for *RFP* (Fig. S1). Colonies were inoculated into 0.5 ml medium and incubated at 30°C with shaking for 2 to 3 h. After harvesting of cells, pellets were suspended in 30 µl 0.2% SDS, heated for 3 min at 95°C, and centrifuged for 1 min at 14,000 × *g*. Three microliters of the supernatant was used as genomic DNA template in a standard 25-µl PCR.

For the analyses of *LEU2* deletions, after transformation with various sgLEU2 gRNA plasmids or control vectors, and with or without donor repair fragments, colonies were patched onto to SD(−Ura) and replica plated onto SD(−Ura) and SD(−Leu) plates. PCR amplification of Leu2^+^ and Leu2^−^ colonies was performed, followed by digestion with *Eco*RI to determine the percentage of colonies that were heterozygous or homozygous for the *leu2Δ* allele.

### Construction of donor healing fragments.

The *rfpΔ* repair fragment targeted deletion of nucleotides 55 to 402 of the *RFP* ORF, including the PAM site located at +132. It was generated by fusion PCR (40). PCR was used to amplify two fragments: one homologous to the 5′ region of *RFP* and the other homologous to the 3′ region. The 5′ fragment also contained a 3′ 20-bp tail that was homologous to the 5′ end of the second fragment (Fig. 2A). These two fragments were mixed, annealed, and then extended. After extension, the full-length “fused” fragment was amplified by PCR. The resulting 593-bp fragment contains a 285-bp arm of upstream sequence homology and a 308-bp arm of downstream sequence homology to regions flanking the DSB. Approximately 1 to 2 µg of this DNA (~5 pmol) was used for transformation of yeast. Recombination with *RFP* results in deletion of an internal 370 bp within the *RFP* ORF, including the PAM site to produce the *rfp*Δ*33-403* allele.

The *LEU2* donor repair fragment was made by annealing and filling in two 60-mer nucleotides that contained 20 bases of sequence complementarity at their 3′ ends. This complementary region included an EcoRI recognition sequence (highlighted in blue in Fig. 7A). The resulting fragment contained 47 bp of homology to sequences flanking the DSB. Three microliters of each oligonucleotide (300 pmol) was annealed and then extended in a 25-µl reaction mixture containing 0.2 mM deoxynucleoside triphosphate (dNTP), buffer, and *Taq* DNA polymerase (Denville Scientific, Inc., United States) and subjected to 25 to 30 cycles of PCR with an extension time of 30 s. Twenty microliters of this reaction mixture was used per yeast transformation as a repair donor fragment (~240 pmol of repair fragment). Recombination with *LEU2* results in replacement of an internal 434-bp fragment within the *LEU2* ORF, including the PAM site, with an EcoRI site to produce the *leu2*Δ*71-505* allele.

## References

[1] Vyas VK, Barrasa MI, Fink GR 2015 A *Candida albicans* CRISPR system permits genetic engineering of essential genes and gene families. Sci Adv 1:e1500248. doi:10.1126/sciadv.1500248. 25977940 PMC4428347

[2] Barrangou R, Fremaux C, Deveau H, Richards M, Boyaval P, Moineau S, Romero DA, Horvath P 2007 CRISPR provides acquired resistance against viruses in prokaryotes. Science 315:1709–1712. doi:10.1126/science.1138140. 17379808

[3] Garneau JE, Dupuis MÈ, Villion M, Romero DA, Barrangou R, Boyaval P, Fremaux C, Horvath P, Magadán AH, Moineau S 2010 The CRISPR/Cas bacterial immune system cleaves bacteriophage and plasmid DNA. Nature 468:67–71. doi:10.1038/nature09523. 21048762

[4] Gasiunas G, Barrangou R, Horvath P, Siksnys V 2012 Cas9-crRNA ribonucleoprotein complex mediates specific DNA cleavage for adaptive immunity in bacteria. Proc Natl Acad Sci U S A 109:E2579–E2586. doi:10.1073/pnas.1208507109. PMC3465414 22949671

[5] Cong L, Ran FA, Cox D, Lin S, Barretto R, Habib N, Hsu PD, Wu X, Jiang W, Marraffini LA, Zhang F 2013 Multiplex genome engineering using CRISPR/Cas systems. Science 339:819–823. doi:10.1126/science.1231143. 23287718 PMC3795411

[6] Jinek M, Chylinski K, Fonfara I, Hauer M, Doudna JA, Charpentier E 2012 A programmable dual-RNA-guided DNA endonuclease in adaptive bacterial immunity. Science 337:816–821. doi:10.1126/science.1225829. 22745249 PMC6286148

[7] Mali P, Yang L, Esvelt KM, Aach J, Guell M, DiCarlo JE, Norville JE, Church GM 2013 RNA-guided human genome engineering via Cas9. Science 339:823–826. doi:10.1126/science.1232033. 23287722 PMC3712628

[8] Donoho G, Jasin M, Berg P 1998 Analysis of gene targeting and intrachromosomal homologous recombination stimulated by genomic double-strand breaks in mouse embryonic stem cells. Mol Cell Biol 18:4070–4078. doi:10.1128/MCB.18.7.4070. 9632791 PMC108991

[9] Storici F, Durham CL, Gordenin DA, Resnick MA 2003 Chromosomal site-specific double-strand breaks are efficiently targeted for repair by oligonucleotides in yeast. Proc Natl Acad Sci U S A 100:14994–14999. doi:10.1073/pnas.2036296100. 14630945 PMC299876

[10] Hsu PD, Scott DA, Weinstein JA, Ran FA, Konermann S, Agarwala V, Li Y, Fine EJ, Wu X, Shalem O, Cradick TJ, Marraffini LA, Bao G, Zhang F 2013 DNA targeting specificity of RNA-guided Cas9 nucleases. Nat Biotechnol 31:827–832. doi:10.1038/nbt.2647. 23873081 PMC3969858

[11] Ryan OW, Skerker JM, Maurer MJ, Li X, Tsai JC, Poddar S, Lee ME, DeLoache W, Dueber JE, Arkin AP, Cate JH 2014 Selection of chromosomal DNA libraries using a multiplex CRISPR system. Elife 3:e03703. doi:10.7554/eLife.03703. PMC4161972 25139909

[12] Min K, Ichikawa Y, Woolford CA, Mitchell AP 2016 Candida albicans gene deletion with a transient CRISPR-Cas9 system. mSphere 1:e00130-16. doi:10.1128/mSphere.00130-16. 27340698 PMC4911798

[13] Marck C, Kachouri-Lafond R, Lafontaine I, Westhof E, Dujon B, Grosjean H 2006 The RNA polymerase III-dependent family of genes in hemiascomycetes: comparative RNomics, decoding strategies, transcription and evolutionary implications. Nucleic Acids Res 34:1816–1835. doi:10.1093/nar/gkl085. 16600899 PMC1447645

[14] DiCarlo JE, Norville JE, Mali P, Rios X, Aach J, Church GM 2013 Genome engineering in Saccharomyces cerevisiae using CRISPR-Cas systems. Nucleic Acids Res 41:4336–4343. doi:10.1093/nar/gkt135. 23460208 PMC3627607

[15] Keppler-Ross S, Noffz C, Dean N 2008 A new purple fluorescent color marker for genetic studies in *Saccharomyces cerevisiae* and *Candida albicans*. Genetics 179:705–710. doi:10.1534/genetics.108.087080. 18493083 PMC2390648

[16] Keppler-Ross S, Douglas L, Konopka JB, Dean N 2010 Recognition of yeast by murine macrophages requires mannan but not glucan. Eukaryot Cell 9:1776–1787. doi:10.1128/EC.00156-10. 20833894 PMC2976302

[17] Gao Y, Zhao Y 2014 Self-processing of ribozyme-flanked RNAs into guide RNAs in vitro and in vivo for CRISPR-mediated genome editing. J Integr Plant Biol 56:343–349. doi:10.1111/jipb.12152. 24373158

[18] Guerrier-Takada C, Gardiner K, Marsh T, Pace N, Altman S 1983 The RNA moiety of ribonuclease P is the catalytic subunit of the enzyme. Cell 35:849–857. doi:10.1016/0092-8674(83)90117-4. 6197186

[19] Schiffer S, Rösch S, Marchfelder A 2002 Assigning a function to a conserved group of proteins: the tRNA 3′-processing enzymes. EMBO J 21:2769–2777. doi:10.1093/emboj/21.11.2769. 12032089 PMC126033

[20] Xie K, Minkenberg B, Yang Y 2015 Boosting CRISPR/Cas9 multiplex editing capability with the endogenous tRNA-processing system. Proc Natl Acad Sci U S A 112:3570–3575. doi:10.1073/pnas.1420294112. 25733849 PMC4371917

[21] Sugawara N, Ira G, Haber JE 2000 DNA length dependence of the single-strand annealing pathway and the role of *Saccharomyces cerevisiae* *RAD59* in double-strand break repair. Mol Cell Biol 20:5300–5309. doi:10.1128/MCB.20.14.5300-5309.2000. 10866686 PMC85979

[22] Moore JK, Haber JE 1996 Cell cycle and genetic requirements of two pathways of nonhomologous end-joining repair of double-strand breaks in *Saccharomyces cerevisiae*. Mol Cell Biol 16:2164–2173. doi:10.1128/MCB.16.5.2164. 8628283 PMC231204

[23] Roth DB, Wilson JH 1986 Nonhomologous recombination in mammalian cells: role for short sequence homologies in the joining reaction. Mol Cell Biol 6:4295–4304. doi:10.1128/MCB.6.12.4295. 3025650 PMC367211

[24] Legrand M, Chan CL, Jauert PA, Kirkpatrick DT 2007 Role of DNA mismatch repair and double-strand break repair in genome stability and antifungal drug resistance in Candida albicans. Eukaryot Cell 6:2194–2205. doi:10.1128/EC.00299-07. 17965250 PMC2168241

[25] Ciudad T, Andaluz E, Steinberg-Neifach O, Lue NF, Gow NA, Calderone RA, Larriba G 2004 Homologous recombination in *Candida albicans*: role of CaRad52p in DNA repair, integration of linear DNA fragments and telomere length. Mol Microbiol 53:1177–1194. doi:10.1111/j.1365-2958.2004.04197.x. 15306020

[26] Wilson RB, Davis D, Enloe BM, Mitchell AP 2000 A recyclable *Candida albicans* *URA3* cassette for PCR product-directed gene disruptions. Yeast 16:65–70. doi:10.1002/(SICI)1097-0061(20000115)16:1<65::AID-YEA508>3.0.CO;2-M. 10620776

[27] Wilson RB, Davis D, Mitchell AP 1999 Rapid hypothesis testing with *Candida albicans* through gene disruption with short homology regions. J Bacteriol 181:1868–1874. 10074081 10.1128/jb.181.6.1868-1874.1999 PMC93587

[28] Wellington M, Rustchenko E 2005 5-Fluoro-orotic acid induces chromosome alterations in *Candida albicans*. Yeast 22:57–70. doi:10.1002/yea.1191. 15635674

[29] Doench JG, Hartenian E, Graham DB, Tothova Z, Hegde M, Smith I, Sullender M, Ebert BL, Xavier RJ, Root DE 2014 Rational design of highly active sgRNAs for CRISPR-Cas9-mediated gene inactivation. Nat Biotechnol 32:1262–1267. doi:10.1038/nbt.3026. 25184501 PMC4262738

[30] Xu H, Xiao T, Chen CH, Li W, Meyer CA, Wu Q, Wu D, Cong L, Zhang F, Liu JS, Brown M, Liu XS 2015 Sequence determinants of improved CRISPR sgRNA design. Genome Res 25:1147–1157. doi:10.1101/gr.191452.115. 26063738 PMC4509999

[31] Gilbert LA, Horlbeck MA, Adamson B, Villalta JE, Chen Y, Whitehead EH, Guimaraes C, Panning B, Ploegh HL, Bassik MC, Qi LS, Kampmann M, Weissman JS 2014 Genome-scale CRISPR-mediated control of gene repression and activation. Cell 159:647–661. doi:10.1016/j.cell.2014.09.029. 25307932 PMC4253859

[32] Farboud B, Meyer BJ 2015 Dramatic enhancement of genome editing by CRISPR/Cas9 through improved guide RNA design. Genetics 199:959–971. doi:10.1534/genetics.115.175166. 25695951 PMC4391549

[33] Radzisheuskaya A, Shlyueva D, Müller I, Helin K 2016 Optimizing sgRNA position markedly improves the efficiency of CRISPR/dCas9-mediated transcriptional repression. Nucleic Acids Res 44:e141. doi:10.1093/nar/gkw583. 27353328 PMC5062975

[34] Smith JD, Suresh S, Schlecht U, Wu M, Wagih O, Peltz G, Davis RW, Steinmetz LM, Parts L, St Onge RP 2016 Quantitative CRISPR interference screens in yeast identify chemical-genetic interactions and new rules for guide RNA design. Genome Biol 17:45. doi:10.1186/s13059-016-0900-9. 26956608 PMC4784398

[35] Bertram G, Swoboda RK, Gooday GW, Gow NA, Brown AJ 1996 Structure and regulation of the *Candida albicans* *ADH1* gene encoding an immunogenic alcohol dehydrogenase. Yeast 12:115–127. 8686375 10.1002/(sici)1097-0061(199602)12:2<115::aid-yea889>3.0.co;2-e

[36] Scott WG, Finch JT, Klug A 1995 The crystal structure of an all-RNA hammerhead ribozyme: a proposed mechanism for RNA catalytic cleavage. Cell 81:991–1002. doi:10.1016/S0092-8674(05)80004-2. 7541315

[37] Nakano S, Chadalavada DM, Bevilacqua PC 2000 General acid-base catalysis in the mechanism of a hepatitis delta virus ribozyme. Science 287:1493–1497. doi:10.1126/science.287.5457.1493. 10688799

[38] Gibson DG, Young L, Chuang RY, Venter JC, Hutchison CA III, Smith HO 2009 Enzymatic assembly of DNA molecules up to several hundred kilobases. Nat Methods 6:343–345. doi:10.1038/nmeth.1318. 19363495

[39] Walther A, Wendland J 2003 An improved transformation protocol for the human fungal pathogen *Candida albicans*. Curr Genet 42:339–343. doi:10.1007/s00294-002-0349-0. 12612807

[40] Higuchi R, Krummel B, Saiki RK 1988 A general method of in vitro preparation and specific mutagenesis of DNA fragments: study of protein and DNA interactions. Nucleic Acids Res 16:7351–7367. doi:10.1093/nar/16.15.7351. 3045756 PMC338413

[41] Darty K, Denise A, Ponty Y 2009 Varna: interactive drawing and editing of the RNA secondary structure. Bioinformatics 25:1974–1975. doi:10.1093/bioinformatics/btp250. 19398448 PMC2712331

[42] Murad AM, Lee PR, Broadbent ID, Barelle CJ, Brown AJ 2000 CIp10, an efficient and convenient integrating vector for *Candida albicans*. Yeast 16:325–327. 10669870 10.1002/1097-0061(20000315)16:4<325::AID-YEA538>3.0.CO;2-#

