# Supplementary figures and images for: Dramatic Improvement of CRISPR/Cas9 Editing in Candida albicans by Increased Single Guide RNA Expression
Source: mSphere. 2017 Apr 19;2(2):e00385-16. doi: 10.1128/mSphere.00385-16 (PMC5397569; doi:10.1128/mSphere.00385-16)

**A**

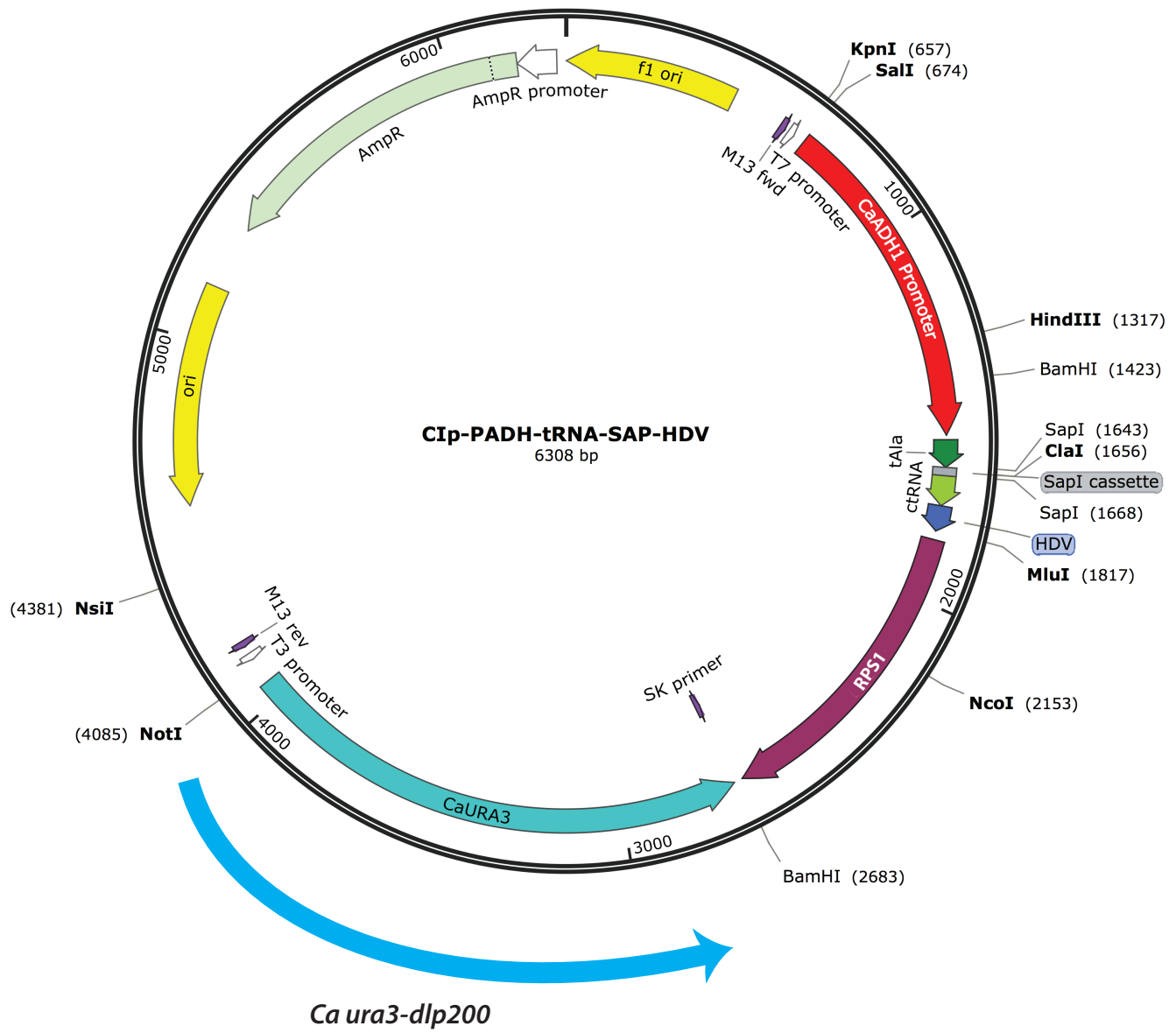

**B**

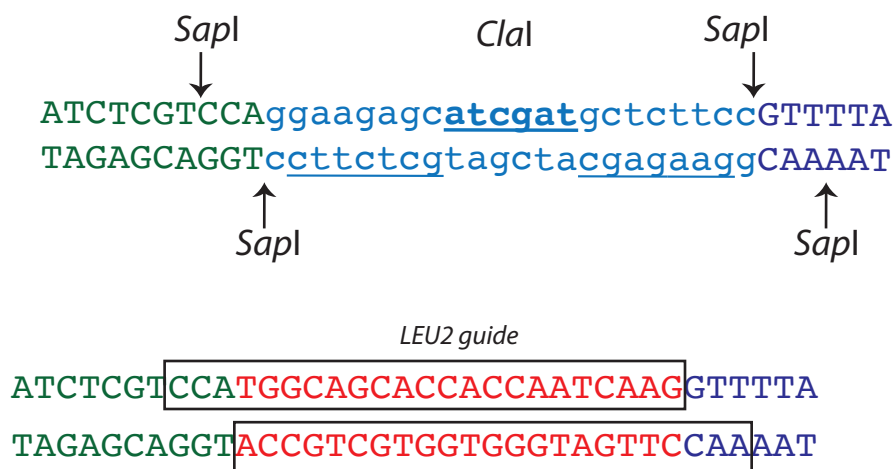

Figure S1 Ng and Dean

Supplement: FIG S1 [file sph002172270sf2.pdf]

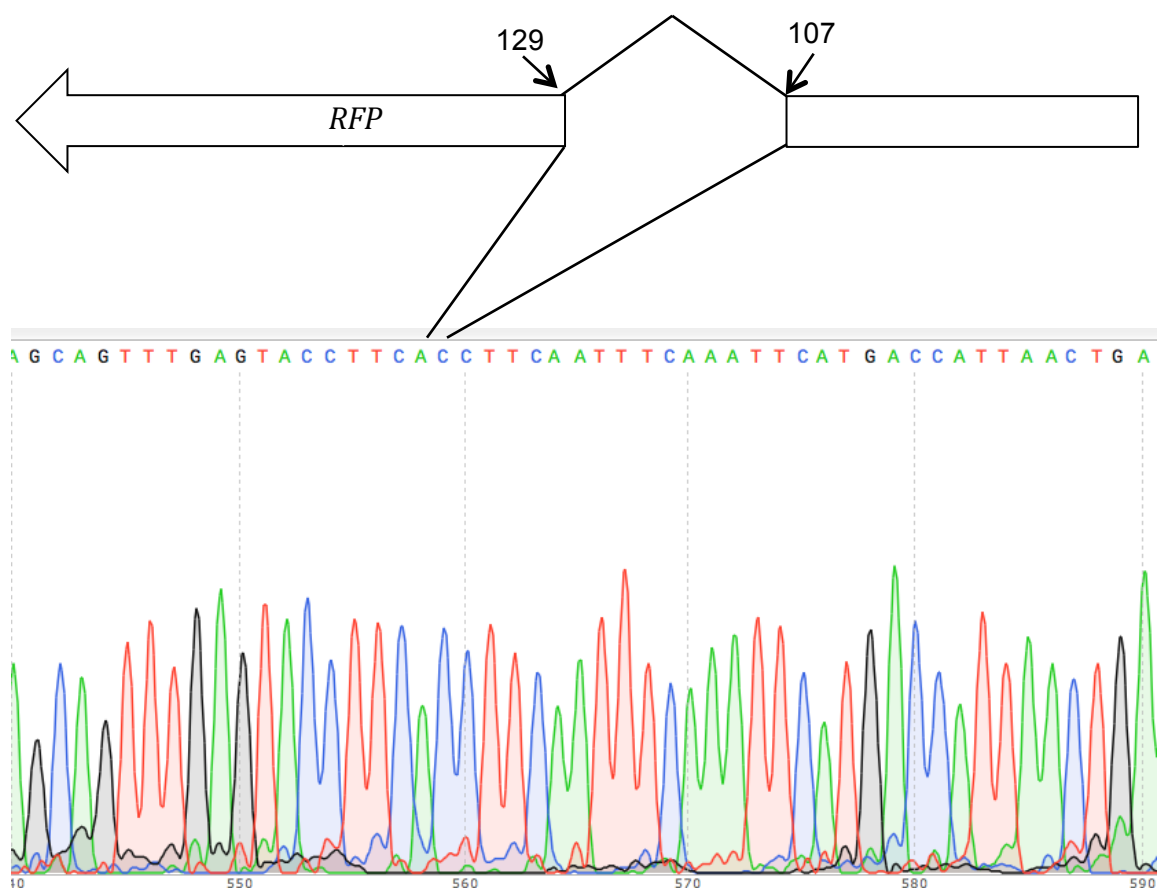

Figure S2. Ng and Dean

Supplement: FIG S2 [file sph002172270sf3.pdf]
