# Supplementary material for: Dramatic Improvement of CRISPR/Cas9 Editing in Candida albicans by Increased Single Guide RNA Expression
Source: mSphere. 2017 Apr 19;2(2):e00385-16. doi: 10.1128/mSphere.00385-16 (PMC5397569; doi:10.1128/mSphere.00385-16)
Supplement: FIG S3 [file sph002172270sf4.pdf]

Basic protocol/time line for single gene knockout

Day 1. Design and order oligonucleotides :

- 3 different gRNA targets (recommended if possible to target one PAM in promoter region upstream initiating ATG) (~23 bases/oligonucleotide- See Figure S1)
- 2 oligonucleotides for donor repair fragment (~ 60 bases/primer- See Figure 7A)
- 2 oligonucleotides (forward and reverse) for genotyping

Day 3-4. Make gRNA plasmid/ healing fragment

- Anneal gRNA oligonucleotides
- Anneal/ fill in donor repair fragment
- Ligate annealed gRNA oligonucleotides into *Sap*I/dephosphorylated cut vector
- Transform *E. coli*
- Put up overnight yeast culture for next day transformation (assume gRNA plasmids are correct)

Day 4-5. Confirm that plasmids are correct/yeast transformation

- Grow *E. coli* / prep gRNA plasmid (6-8 hours incubation @ 37°)
- Dilute yeast culture 1:20 for transformation
- Check gRNA expression for loss of *Cla*I *site*
- Digest w/ *Stu*I for yeast transformation
- Transform yeast (or do next day)

Day 6/7- transform *C. albicans* (best strain is new HNY31… *ura3*∆/∆ *his1*∆/∆ *arg4*∆/∆ *leu2*∆/∆ *ENO1/eno1*∆*::CaCas9*

Day 9/10

- Assay Ura3$^+$ transformants by PCR.
- If correct, streak for single colonies, recheck, then **STOCK 2-3 strains.**

Figure S3 Ng and Dean
