# Supplementary material for: Dramatic Improvement of CRISPR/Cas9 Editing in Candida albicans by Increased Single Guide RNA Expression
Source: mSphere. 2017 Apr 19;2(2):e00385-16. doi: 10.1128/mSphere.00385-16 (PMC5397569; doi:10.1128/mSphere.00385-16)
Supplement: TABLE S1 [file sph002172270st1.pdf]

Name	Primers	Sequence (5` -> 3')	Key Features	Key Restriction Sites
gRFP-2		GGTGAAGGTAGACCATATGA	*RFP* guide RNA	
gLEU2-1		TGGCAGCACCACCAATCAAG	*LEU2* gRNA	
P_{ADH}HHgRFP<br>HDV		GTCGACATGTCAAAGGATTCAACGGTTGAGATGGAGCCGT<br>CAAATATCCAAAACACTCTTCTTTCAACTAATTTGCCGTAAA<br>CTATCTCCATCGTACGATTAAGATAAAAAAAAACAATTATTT<br>CTTACAATATTTGATAGAGACCCAATGCAAAGCCAGCGAG<br>AAAGAGGAGTATTGGCATTGTTGGGTAGTAGATGAATGAG<br>TAATCTTCTAGCCAAAACAAAATAAACCACAAGAACAGTAG<br>GCAAAACTGAGTACTTGATAGCGCTTGTAGTCTTTGTATTT<br>AAAAAAACCTAATTGTCCAAAAATTGAAAATCATGAGTTGC<br>AAATTTGTTGAAATCACCAGGTAAATAGATCATTTAGCTATA<br>TCGCCTTATCATTCTTCGTACAAGAATTATACACAAATACC<br>ACCACCACACCACCCAGAAATTTTACACGTGTTTTCTAAAT<br>TATTACTTTCCGTGGCCAATCAAATTGAAATTTTTCTTGTTC<br>GAAACGGCAGTGCCTCAAACATTATAGTAGCAAGTATTGC<br>CTAAAGAGTTTCCGTCTCGTGCCCCCCCCCTATCCCCTTTT<br>GTAATTTGCCAAAATAGCTCAAAAAGGATAATCCCGCACTT<br>TTTTTTTCCGAGTTCTCGTCATGCAAGCAAGCTTATTCAGA<br>ATTTTCAGAGGTGCGAGTCTCACCACTACCACTGCAGCTG<br>CATCTTTTACAGCAACAGCAACAGCAGGTGCCACCACGGC<br>AAAGACATTGTCTGGATCCACTGTGCTCCGAAAACTGTATA<br>AAAGGACCTATTCATCCCTGGTCTTATCTTCTCCAGAATTA<br>TTTTTTTTTCATCAGTTTAACAACAACAAACGTTATTGTCAT<br>ACAACAACAACAACAAATACAAAAACAATTagatcTTTCACCct<br>gatgagtccgtgaggacgaaacgagtaagctcgtcGGTGAAGGTAGACCA<br>TATGAgttttagagctagaaatagcAagttaaaataaggctagtccgttatcaacttg<br>aaaaagtggcaccgagtcggtgcttttGGCCGGCATGGTCCCAGCCTC<br>CTCGCTGGCGCCGGCTGGGCAACATGCTTCGGCATGGCG<br>AATGGGACacgcgt	Red:*ADH1* Promoter;<br>Green: Hammer-head;<br>Blue: 20bp gRFP; Purple:<br>Cas9 binding Motif;<br>Orange: HDV	*Sal*I, *Bgl*II,<br>*Mlu*I

P_{ADH}tAgRFP HDV		GTCGACATGTCAAAGGATTCAACGGTTGAGATGGAGCCGT<br>CAAATATCCAAAACACTCTTCTTTCAACTAATTTGCCGTAAA<br>CTATCTCCATCGTACGATTAAGATAAAAAAAAACAATTATTT<br>CTTACAATATTTGATAGAGACCCAATGCAAAGCCAGCGAG<br>AAAGAGGAGTATTGGCATTGTTGGGTAGTAGATGAATGAG<br>TAATCTTCTAGCCAAAACAAAATAAACCACAAGAACAGTAG<br>GCAAAACTGAGTACTTGATAGCGCTTGTAGTCTTTGTATTT<br>AAAAAAACCTAATTGTCCAAAAATTGAAAATCATGAGTTGC<br>AAATTTGTTGAAATCACCAGGTAAATAGATCATTTAGCTATA<br>TCGCCTTATCATTCTTCGTACAAGAATTATACACAAATACC<br>ACCACCACACCACCCAGAAATTTTACACGTGTTTTCTAAAT<br>TATTACTTTCCGTGGCCAATCAAATTGAAATTTTTCTTGTTC<br>GAAACGGCAGTGCCTCAAACATTATAGTAGCAAGTATTGC<br>CTAAAGAGTTTCCGTCTCGTGCCCCCCCCCTATCCCCTTTT<br>GTAATTTGCCAAAATAGCTCAAAAAGGATAATCCCGCACTT<br>TTTTTTTCCGAGTTCTCGTCATGCAAGCAAGCTTATTCAGA<br>ATTTTCAGAGGTGCGAGTCTCACCACTACCACTGCAGCTG<br>CATCTTTTACAGCAACAGCAACAGCAGGTGCCACCACGGC<br>AAAGACATTGTCTGGATCCACTGTGCTCCGAAAACTGTATA<br>AAAGGACCTATTCATCCCTGGTCTTATCTTCTCCAGAATTA<br>TTTTTTTTTCATCAGTTTAACAACAACAAACGTTATTGTCAT<br>ACAACAACAACAACAAATACAAAAACAAAGAGCTCAAAACG<br>GGCGTGTGGCGTAGTTGGTAGCGCGTTCCCTTAGCATGG<br>GAAAGGTCATGAGTTCGACTCTTATCTCGTCCAGGTGAAG<br>GTAGACCATATGAGTTTTAGAGCTAGAAATAGCAAGTTAAA<br>ATAAGGCTAGTCCGTTATCAACTTGAAAAAGTGGCACCGA<br>GTCGGTGCTTTTGGCCGGCATGGTCCCAGCCTCCTCGCT<br>GGCGCCGGCTGGGCAACATGCTTCGGCATGGCGAATGGG<br>ACACGCGT	Red:*ADH1* Promoter; Green: tRNA^{Ala}; Blue: 20bp gRFP; Purple: Cas9 binding Motif; Orange: HDV	*Sal*I, SacI, MluI
PtAgRFPHD V		gtcgacgctctcccttatgcgactcctgcattaggaagcagcccagtagtaggttgagg<br>ccgttgagcaccgccgccgcaaggaatggtgcatgcaaggagatggcgcccaaca<br>gtcccccggccacggggcctgccaccatacccacgccgaaacaagcgctcatgag<br>cccgaagtggcgagcccgatcttccccatcggtgatgtcggcgatataggcgccagc<br>aaccgcacctgtggcgccggtgatgccggccacgatgcgtccggcgtagaggatca<br>gcggccGATCTcaaaacGGGCGTGTGGCGTAGTTGGTAGCGC<br>GTTCCCTTAGCATGGGAAAGGTCATGAGTTCGACTCTTAT	Grey: YPB vector; Green: tRNA^{Ala} ; Blue: 20bp gRFP; Purple: Cas9 binding Motif; Orange: HDV	*Sal*I, *Mlu*I

		CTCGTCCAGGTGAAGGTAGACCATATGAGTTTTAGAGCTA GAAATAGCAAGTTAAAATAAGGCTAGTCCGTTATCAACTTG AAAAAGTGGCACCGAGTCGGTGCTTTTGGCCGGCATGGT CCCAGCCTCCTCGCTGGCGCCGGCTGGGCAACATGCTTC GGCATGGCGAATGGGACACGCGT		
P_{SNR52}gRFP	984/1002	gtcgacAAGTGATTAGACTTAGTCCGTTCAAATCAAGCACAA CTCTGTTCATTGTTTCAACAAGAATTAATTCAAAAACAGGTT CGGTGCATAATTTGCAAAAAAATATTGCAGCTTCTGTGGCT CGAACACAGTACCTCCAGATTTCAGGTTTGAAATACTTCAG TCTGACGCTCTCCCAGATGAGCTAAAGCTGCAATAAGAAA ACCCACGCCGGGATTCGAACCCGGAATCCTTTGATTAGAA GTCAAAAGCGATAACCATTTCGCCACGCAGGCCTACTTGA TGGGTTTGTAAATGGTCTACTTTTTCAGACCTAACAGAAAT TTTAATGAAAGTCATATTCTTATACAATAAAACTGTGTCATA AAAGCAGATATTCGACTTTCGTAGATTATATAGGACCCAAG AACTAAAATTTAATGCCATATTATGCATTTTTAATCTGTAAA AGTGTTGTTTCCAACCTATCACAAGTACGTTCTTGTAACTT GTGTTTGTAGGGTTGCAAATGAATCATAACAACATCTCAAC AGAACATGTATAGCAAAGCTTAGTATAAAATCAGTGTTTTG AGAGGCAATCCAAGAATGTTTACATCAAAGTTTCAATAAAT ATCGACCGAAACTGAAAATCTTTTTAGGTTATTGTTCACTTT TTTGTAAATATTTAAACATTTTTTGGACCTAAAAAAATACAA ACACCAATTACGTACCAAGAAGCATCTAATCAACTCCCAGA TCACCACTATACATTTAAAAGTCATTGGTCAATAACTATACT CGAGTATTGCCTCATCAAAGAAACAATCAAATATTATAGAT ACTCACTCCATCACGTGATAATTTCACTGGTATGGAAAAGT GGAAAATTTTATAAAAAAAAATTTGATGCCTTTGGCATAGC TGAAACTTCGGCCCAATAGGATTGGAGAATATGTTTTCGCA GCGTTCTTACAATTAAATTGTGGTGGAAGTTCGAGACTTGC GTAAACTATTTTTAATTTgGTGAAGGTAGACCATATGAgttttag agctagaaatagcAagttaaaataaggctagtccgttatcaacttgaaaaagtggca ccgagtcggtgcttttacgcgt	Red: *SNR52* Promoter; Blue: 20bp gRFP; Purple: Cas9 binding motif	*Sal*I, *Mlu*I
P_{ADH}tA"SapI cassette"HDV		GTCGACATGTCAAAGGATTCAACGGTTGAGATGGAGCCGT CAAATATCCAAAACACTCTTCTTTCAACTAATTTGCCGTAAA CTATCTCCATCGTACGATTAAGATAAAAAAAAACAATTATTT	Red:*ADH1* Promoter; Green tRNA^{Ala} ; Blue: *Sap*I Cassette; Purple:	*Sal*I,*Sac*I, *Sap*I, *Cla*I,

		CTTACAATATTTGATAGAGACCCAATGCAAAGCCAGCGAG AAAGAGGAGTATTGGCATTGTTGGGTAGTAGATGAATGAG TAATCTTCTAGCCAAAACAAAATAAACCACAAGAACAGTAG GCAAAACTGAGTACTTGATAGCGCTTGTAGTCTTTGTATTT AAAAAAACCTAATTGTCCAAAAATTGAAAATCATGAGTTGC AAATTTGTTGAAATCACCAGGTAAATAGATCATTTAGCTATA TCGCCTTATCATTCTTCGTACAAGAATTATACACAAATACC ACCACCACACCACCCAGAAATTTTACACGTGTTTTCTAAAT TATTACTTTCCGTGGCCAATCAAATTGAAATTTTTCTTGTTC GAAACGGCAGTGCCTCAAACATTATAGTAGCAAGTATTGC CTAAAGAGTTTCCGTCTCGTGCCCCCCCCCTATCCCCTTTT GTAATTTGCCAAAATAGCTCAAAAAGGATAATCCCGCACTT TTTTTTTCCGAGTTCTCGTCATGCAAGCAAGCTTATTCAGA ATTTTCAGAGGTGCGAGTCTCACCACTACCACTGCAGCTG CATCTTTTACAGCAACAGCAACAGCAGGTGCCACCACGGC AAAGACATTGTCTGGATCCACTGTGCTCCGAAAACTGTATA AAAGGACCTATTCATCCCTGGTCTTATCTTCTCCAGAATTA TTTTTTTTTCATCAGTTTAACAACAACAAACGTTATTGTCAT ACAACAACAACAACAAATACAAAAACAAAGAGCTCAAAACG GGCGTGTGGCGTAGTTGGTAGCGCGTTCCCTTAGCATGG GAAAGGTCATGAGTTCGACTCTTATCTCGTCCAGGAAGAG CATCGATGCTCTTCCGTTTTAGAGCTAGAAATAGCAAGTTA AAATAAGGCTAGTCCGTTATCAACTTGAAAAAGTGGCACC GAGTCGGTGCTTTTGGCCGGCATGGTCCCAGCCTCCTCG CTGGCGCCGGCTGGGCAACATGCTTCGGCATGGCGAATG GGAC	Cas9 binding Motif; Orange: HDV	*Sap*I,
P_{ADH}RFP	701/629	GTGCTCGGTACCGTCGACATGTCAAAGGATTCAACGGTTG AGATGGAGCCGTCAAATATCCAAAACACTCTTCTTTCAACT AATTTGCCGTAAACTATCTCCATCGTACGATTAAGATAAAA AAAAACAATTATTTCTTACAATATTTGATAGAGACCCAATGC AAAGCCAGCGAGAAAGAGGAGTATTGGCATTGTTGGGTAG TAGATGAATGAGTAATCTTCTAGCCAAAACAAAATAAACCA CAAGAACAGTAGGCAAAACTGAGTACTTGATAGCGCTTGT AGTCTTTGTATTTAAAAAAACCTAATTGTCCAAAAATTGAAA ATCATGAGTTGCAAATTTGTTGAAATCACCAGGTAAATAGA TCATTTAGCTATATCGCCTTATCATTCTTCGTACAAGAATTA		*Kpn*I,*Bgl*II, *Mlu*I

		TACACAAATACCACCACCACACCACCCAGAAATTTTACACG<br>TGTTTTCTAAATTATTACTTTCCGTGGCCAATCAAATTGAAA<br>TTTTTCTTGTTCGAAACGGCAGTGCCTCAAACATTATAGTA<br>GCAAGTATTGCCTAAAGAGTTTCCGTCTCGTGCCCCCCCC<br>CTATCCCCTTTTGTAATTTGCCAAAATAGCTCAAAAAGGAT<br>AATCCCGCACTTTTTTTTTCCGAGTTCTCGTCATGCAAGCA<br>AGCTTATTCAGAATTTTCAGAGGTGCGAGTCTCACCACTAC<br>CACTGCAGCTGCATCTTTTACAGCAACAGCAACAGCAGGT<br>GCCACCACGGCAAAGACATTGTCTGGATCCACTGTGCTCC<br>GAAAACTGTATAAAAGGACCTATTCATCCCTGGTCTTATCT<br>TCTCCAGAATTATTTTTTTTTCATCAGTTTAACAACAACAAA<br>CGTTATTGTCATACAACAACAACAACAAATACAAAAACAAT<br>Tagatctatggtttcaaaaggtgaagaagataatatggctattattaaagaatttatgag<br>atttaaagttcatatggaaggttcagttaatggtcatgaatttgaaattgaaggtgaaggt<br>gaaggtagaccatatgaaggtactcaaactgctaaattgaaagttactaaaggtggtc<br>cattaccatttgcttgggatattttgtcaccacaatttatgtatggttcaaaagcttatgttaa<br>acatccagctgatattccagattatttaaaattgtcatttccagaaggttttaaatgggaaa<br>gagttatgaattttgaagatggtggtgttgttactgttactcaagattcatcattacaagatg<br>gtgaatttatttataaagttaaattgagaggtactaattttccatcagatggtccagttatgc<br>aaaaaaaaactatgggttgggaagcttcatcagaaagaatgtatccagaagatggtg<br>ctttaaaaggtgaaattaaacaaagattgaaattaaaagatggtggtcattatgatgctg<br>aagttaaaactacttataaagctaaaaaaccagttcaattaccaggtgcttataatgtta<br>atattaaattggatattacttcacataatgaagattatactattgttgaacaatatgaaaga<br>gctgaaggtagacattcaactggtggtatggatgaattatataaataaACGCGT		
P_{ADH}RFP<br>"Full Length"	920/629	GCTTATTCAGAATTTTCAGAGGTGCGAGTCTCACCACTACC<br>ACTGCAGCTGCATCTTTTACAGCAACAGCAACAGCAGGTG<br>CCACCACGGCAAAGACATTGTCTGGATCCACTGTGCTCCG<br>AAAACTGTATAAAAGGACCTATTCATCCCTGGTCTTATCTT<br>CTCCAGAATTATTTTTTTTTCATCAGTTTAACAACAACAAAC<br>GTTATTGTCATACAACAACAACAACAAATACAAAAACAATTa<br>gatctatggtttcaaaaggtgaagaagataatatggctattattaaagaatttatgagatt<br>taaagttcatatggaaggttcagttaatggtcatgaatttgaaattgaaggtgaaggtga<br>aggtagaccatatgaaggtactcaaactgctaaattgaaagttactaaaggtggtccat<br>taccatttgcttgggatattttgtcaccacaatttatgtatggttcaaaagcttatgttaaaca<br>tccagctgatattccagattatttaaaattgtcatttccagaaggttttaaatgggaaaga<br>gttatgaattttgaagatggtggtgttgttactgttactcaagattcatcattacaagatggt		*Bam*HI,*Mlu*I

		gaatttatttataaagttaaattgagaggtactaattttccatcagatggtccagttatgca aaaaaaaaactatgggttgggaagcttcatcagaaagaatgtatccagaagatggtgc tttaaaaggtgaaattaaacaaagattgaaattaaaagatggtggtcattatgatgctg aagttaaaactacttataaagctaaaaaaccagttcaattaccaggtgcttataatgtta atattaaattggatattacttcacataatgaagattatactattgttgaacaatatgaaaga gctgaaggtagacattcaactggtggtatggatgaattatataaataaACGCGT		
RFP fusion fragment-top	920/954	GCTTATTCAGAATTTTCAGAGGTGCGAGTCTCACCACTACC ACTGCAGCTGCATCTTTTACAGCAACAGCAACAGCAGGTG CCACCACGGCAAAGACATTGTCTGGATCCACTGTGCTCCG AAAACTGTATAAAAGGACCTATTCATCCCTGGTCTTATCTT CTCCAGAATTATTTTTTTTTCATCAGTTTAACAACAACAAAC GTTATTGTCATACAACAACAACAACAAATACAAAAACAATTa gatctatggtttcaaaaggtgaagaagataatatggcCCATCAGATGGTCCA GTT	Red: sequence homology between oligonucleotides	
RFP fusion fragment bottom	953/629	GGCCCTACGCGTttatttatataattcatccataccaccagttgaatgtctaccttc agctctttcatattgttcaacaatagtataatcttcattatgtgaagtaatatccaatttaatat taacattataagcacctggtaattgaactggtttttagctttataagtagttttaacttcagc atcataatgaccaccatcttttaatttcaatctttgtttaatttcaccttttaaagcaccatcttc tggatacattctttctgatgaagcttcccaacccatagttttttttgcataactggaccatct gatgg	Red: sequence homology between oligonucleotides	
RFP Healing Fragment	920/954	GCTTATTCAGAATTTTCAGAGGTGCGAGTCTCACCACTACC ACTGCAGCTGCATCTTTTACAGCAACAGCAACAGCAGGTG CCACCACGGCAAAGACATTGTCTGGATCCACTGTGCTCCG AAAACTGTATAAAAGGACCTATTCATCCCTGGTCTTATCTT CTCCAGAATTATTTTTTTTTCATCAGTTTAACAACAACAAAC GTTATTGTCATACAACAACAACAACAAATACAAAAACAATTa gatctatggtttcaaaaggtgaagaagataatatggcccatcagatggtccagttatgc aaaaaaaaactatgggttgggaagcttcatcagaaagaatgtatccagaagatggtg ctttaaaaggtgaaattaaacaaagattgaaattaaaagatggtggtcattatgatgctg aagttaaaactacttataaagctaaaaaaccagttcaattaccaggtgcttataatgtta atattaaattggatattacttcacataatgaagattatactattgttgaacaatatgaaaga gctgaaggtagacattcaactggtggtatggatgaattatataaataaACGCGTA GGGCC	Red: sequence homology between oligonucleotides	

LEU2 healing oligo- top	976	ACTGTCTTGCCTGGTGACCATGTCGGTACCGAAATTGTCA ATGAAGCgaattcCTGTTGA	Purple, Red: homologous sequence. Blue- *Eco*RI site	
LEU2 healing oligo-bottom	977	AGCCATAAATGCTGCCATACGAGTGATACGAGTAACTTCG TCAACAGgaattcGCTTCAT	Purple, Red: homologous sequence. Blue- *Eco*RI site	*Eco*RI
LEU2 healing fragment	976+977	ACTGTCTTGCCTGGTGACCATGTCGGTACCGAAATTGTCA ATGAAGCGAATTCCTGTTGACGAAGTTACTCGTATCACTCG TATGGCAGCATTTATGGCT	Blue- *Eco*RI site	*Eco*RI
